# The YTH domain‐containing protein family: Emerging players in immunomodulation and tumour immunotherapy targets

**DOI:** 10.1002/ctm2.1784

**Published:** 2024-08-12

**Authors:** Fenghe Li, Chong Zeng, Jie Liu, Lei Wang, Xiaorui Yuan, Li Yuan, Xiaomeng Xia, Wei Huang

**Affiliations:** ^1^ Department of Gynaecology and Obstetrics Second Xiangya Hospital Central South University Changsha China; ^2^ Department of Respiratory and Critical Care Medicine The Seventh Affiliated Hospital, Hengyang Medical School, University of South China Changsha Hunan China; ^3^ Department of Pathology The Affiliated Changsha Central Hospital, Hengyang Medical School, University of South China Changsha Hunan China; ^4^ NHC Key Laboratory of Carcinogenesis and the Key Laboratory of Carcinogenesis and Cancer Invasion of the Chinese Ministry of Education Cancer Research Institute, School of Basic Medical Science, Central South University Changsha Hunan China; ^5^ Department of Nuclear Medicine The Third Xiangya Hospital Central South University Changsha Hunan China; ^6^ Department of Oncology Xiangya Hospital Central South University Changsha China; ^7^ National Clinical Research Center of Geriatric Disorders Xiangya Hospital Central South University Changsha China; ^8^ Research Center of Carcinogenesis and Targeted Therapy Xiangya Hospital Central South University Changsha China

**Keywords:** anti‐tumour immunity, immunoregulation, m6A methylation, YTH domain‐containing protein family

## Abstract

**Background:**

The modification of N6‐methyladenosine (m6A) plays a pivotal role in tumor by altering both innate and adaptive immune systems through various pathways, including the regulation of messenger RNA. The YTH domain protein family, acting as “readers” of m6A modifications, affects RNA splicing, stability, and immunogenicity, thereby playing essential roles in immune regulation and antitumor immunity. Despite their significance, the impact of the YTH domain protein family on tumor initiation and progression, as well as their involvement in tumor immune regulation and therapy, remains underexplored and lacks comprehensive review.

**Conclusion:**

This review introduces the molecular characteristics of the YTH domain protein family and their physiological and pathological roles in biological behavior, emphasizing their mechanisms in regulating immune responses and antitumor immunity. Additionally, the review discusses the roles of the YTH domain protein family in immune‐related diseases and tumor resistance, highlighting that abnormal expression or dysfunction of YTH proteins is closely linked to tumor resistance.

**Key points:**

- This review provides an in‐depth understanding of the YTH domain protein family in immune regulation and antitumor immunity, suggesting new strategies and directions for immunotherapy of related diseases.
- These insights not only deepen our comprehension of m6A modifications and YTH protein functions but also pave the way for future research and clinical applications.

## Graphical abstract

- The YTH domain protein family regulates innate and adaptive immunity through regulating multiple immune cells and plays a role in anti‐tumour immunity.
- The YTH domain protein family influences the tumour immune microenvironment by targeting YAP and metabolic mechanisms, promoting immune escape and metastasis.
- The YTH domain protein family is a potential target for immune‐related diseases and tumour immunotherapy.

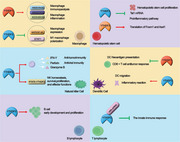

## 1 INTRODUCTION

Epigenetics involves inheritable and reversible changes in gene expression without DNA sequence changes, gaining significant focus recently. M6A methylation, key in eukaryotic cells, affects organism development and disease progression by regulating gene expression post‐transcriptionally.^1^, ^2^ M6A methylation at adenine's sixth nitrogen dominates in mRNA and long noncoding RNA (lncRNA) with high prevalence in mRNA's stop codons, 3′‐untranslated regions (3′‐UTRs) and long internal exons.^3^, ^4^, ^5^ Immune suppression may be reversed by epigenetic treatments, such as histone deacetylase inhibitors and DNA methyltransferase inhibitors.^6^ In addition to alterations to DNA and histones, post‐transcriptional RNA modifications have become a noteworthy epigenetic mechanism governing gene expression,^1^, ^7^ capturing considerable research interest. This modification represents a dynamic biological process involving three distinct components: ‘writers’, ‘erasers’ and ‘readers’.^8^, ^9^ Methyltransferase complexes, often referred to as ‘writers’, are in charge of causing the m6A alteration to occur. This large protein complex includes key components such as methyltransferase‐like protein 3 (METTL3), methyltransferase‐like protein 14 (METTL14), Wilms tumour 1‐associated protein (WTAP), Vir‐like m6A methyltransferase associated (VIRMA), RNA binding motif protein 15 (RBM15) and zinc finger CCCH‐type containing 13 (ZC3H13). M6A is demethylated by demethylases (erasers), such as fat mass and obesity‐associated protein (FTO) and AlkB homolog 5 (ALKBH5), which transform it into N6‐hydroxymethyladenosine and N6‐adenosine, eventually hydrolysing to adenosine. M6A methylation reader proteins, also known as readers, are RNA‐binding proteins that take role in RNA splicing, regulation, stability, translation, nuclear export and destruction. They also identify modification sites on downstream target genes.^10^, ^11^, ^12^ The primary ‘readers’ include the YT521‐B homology (YTH) domain family, insulin‐like growth factor 2 mRNA binding proteins (IGF2BPs), eIF3 and heterogeneous nuclear ribonucleoproteins (HNRNPs).^13^, ^14^ RNA m6A modifications dynamically and reversibly form through the involvement of methyltransferases and demethylases. Proteins that selectively recognise m6A modifications have the capacity to bind with m6A RNA and impart distinct functions.^15^ Dysregulation of m6A methylation is associated with numerous human diseases, highlighting its potential in therapeutic applications for various cancers and diseases. Studies indicate that m6A modifications regulate both innate and adaptive immunity through diverse mechanisms. For example, m6A strictly regulates various innate immune reactions, modulates interferon responses on host immune‐related molecules, influences immune cell differentiation and affects the production of inflammatory cytokines and other immune responses induced by viral infections.^16^, ^17^, ^18^, ^19^ Immune components within the tumour microenvironment (TME) coordinate tumour immunity, significantly affecting tumour occurrence, progression, metastasis and treatment response.^20^, ^21^, ^22^, ^23^, ^24^, ^25^, ^26^, ^27^ M6A modification is essential for controlling the tumour immune microenvironment (TIME) in addition to controlling the activity of transcription factors, non‐coding RNA molecules and signalling pathways linked to carcinogenic consequences.^28^, ^29^, ^30^ Additionally, m6A modifications identify and respond to intruding pathogens and modify external or aberrant endogenous RNAs.^31^, ^32^, ^33^ As understanding of m6A modification mechanisms and regulatory functions deepens, the functional ‘readers’ of the YTH domain‐containing protein family have gained increasing attention in immunoregulation and anti‐tumour immunity.

The YTH domain category in humans is made up of the following five proteins: YTHDF1, YTHDF2, YTHDF3, YTHDC1 and YTHDC2. YTHDF1–3 and YTHDC2 are primarily located in the cytoplasm, whereas YTHDC1 is found in the nucleus.^15^ Each protein has distinct functions with limited redundancy, guiding different complexes to regulate RNA signalling pathways.^15^ Despite sharing highly conserved YTH domains, these proteins exhibit varied and interrelated functional scopes. The critical roles these homologous proteins play in cellular biological processes are highlighted by their conserved YTH structure. Nearly every phase of m6A‐modified RNA, including RNA folding, splicing, translation and metabolism, is regulated by YTH proteins.^15^ Additionally, new research has shown that YTH proteins control the activities of non‐coding RNAs, suggesting their significance in controlling non‐coding RNA regulation to modify cellular processes.^34^ Due to their diverse roles in RNA regulation, dysfunctions in these YTH proteins contribute significantly to various cancers. The YTH protein family also plays significant roles in immune regulation. By promoting the translation of genes linked to immunity, YTHDF1 improves the immune system's response.^35^ YTHDF2 influences the development and roles of immune cells by controlling mRNA stability in immune cells.^36^ Through their control on the expression of immunity‐related genes, YTHDC1 and YTHDC2 participate in immunological responses.^37^, ^38^ The extensive roles of these proteins in RNA regulation make them potential therapeutic targets, particularly in tumour immunotherapy and immune‐related diseases. In conclusion, the human YTH domain protein family plays multifaceted roles in cell biology and disease. Understanding their specific mechanisms and functions can aid in developing more effective therapies, particularly in cancer and immunotherapy.

In recent years, YTH family proteins have emerged as significant players in the field of m6A modification epigenetics study. While there is ongoing dispute on the roles that various YTH proteins play in carcinogenesis, numerous studies have shown that these proteins play diverse roles in the process. Additionally, YTH proteins hold significant importance in tumour immunity, tumour metabolism, drug resistance and other immune‐related diseases. These fields still lack enough in‐depth investigation, despite their importance. Comprehensive reviews that examine these proteins’ interactions with the TME, effects on immune responses and consequences for disease outcomes are currently lacking. This review first introduces the structure, function and physiological roles of YTH family proteins. It then focuses on how the YTH domain protein family regulates immune responses and participates in anti‐tumour immune processes, summarising their roles in different diseases. Overall, the human YTH domain protein family exhibits multifaceted roles in cell biology and disease. Understanding their specific mechanisms and functions can aid in developing more effective therapies, particularly in cancer and immunotherapy.

## 2 STRUCTURE, FUNCTION, PHYSIOLOGICAL AND PATHOLOGICAL ROLES OF THE YTH DOMAIN PROTEIN FAMILY

**FIGURE 1:**
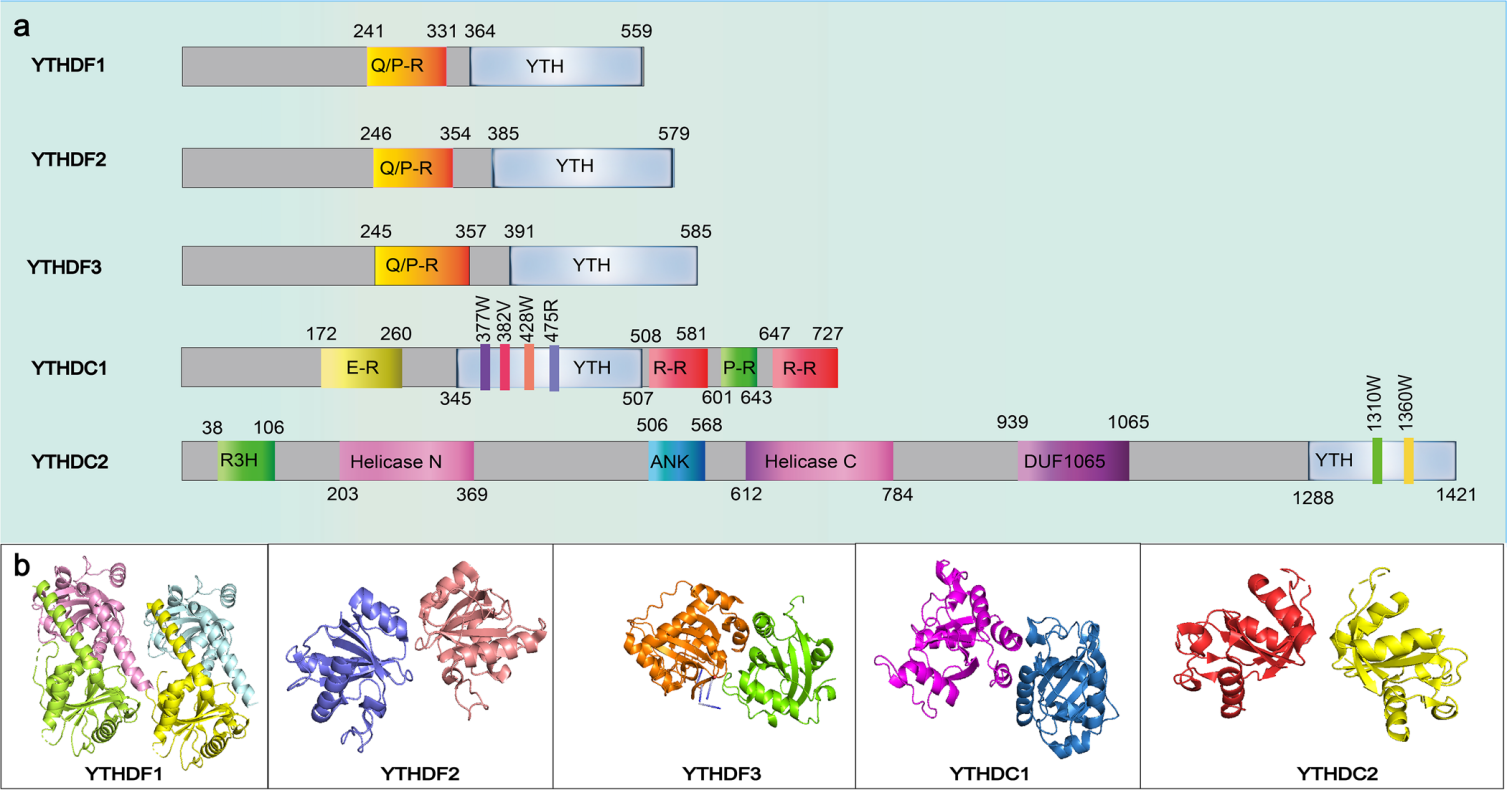
The structure of human YTH domain‐containing proteins. (A) The domain composition of YTHDF1–3 and YTHDC1–2. The five proteins YTHDF1, YTHDF2, YTHDF3, YTHDC1 and YTHDC2 share a highly conserved YTH domain (blue). (B) 3D structure of the YTH domain protein family: crystal structure of the YTHDF1 YTH domain, crystal structure of the N6‐methyladenosine RNA reader YTHDF2, crystal structure of the YTHDF3 YTH domain in complex with m6A RNA, crystal structure of the YTHDC1 T379V mutant and crystal structure of the human YTHDC2 YTH domain.

**FIGURE 2:**
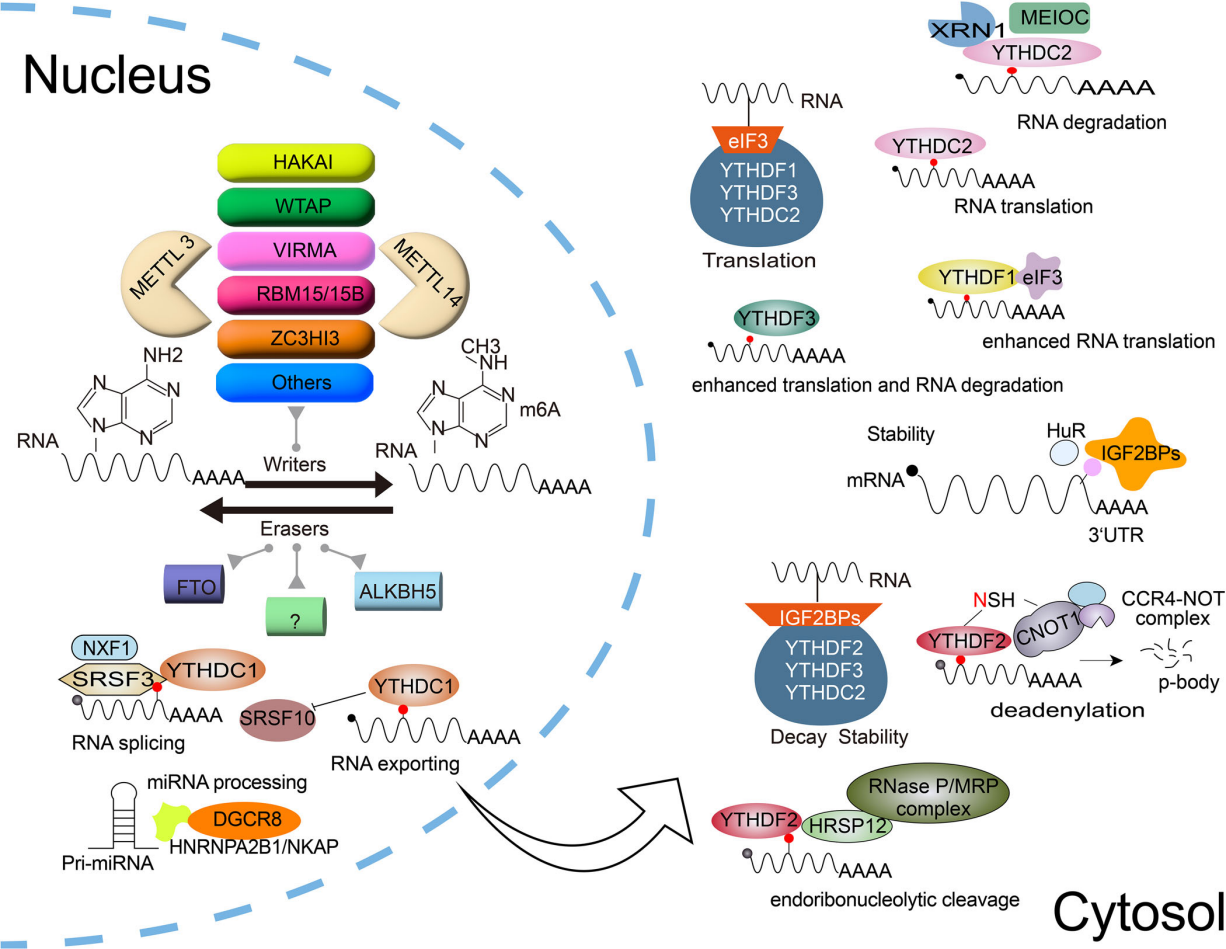
M6A modification‐related protein function and localisation in cells. In the cytoplasm, YTHDF1 promotes the translation of m6A‐modified mRNA; YTHDF2 accelerates the degradation of target mRNA; YTHDF3 can cooperate with YTHDF1 to promote mRNA translation and accelerate the degradation of m6A‐modified transcripts mediated by YTHDF2. In the nucleus, YTHDC1 regulates splicing events by promoting SRSF3 or inhibiting SRSF10 and can interact with nuclear RNA export factor 1 (NXF1) to facilitate mRNA export from the nucleus; YTHDC2 is an RNA helicase whose helicase domain also contributes to RNA binding, involved in regulating mRNA translation or degradation. EIF3, a eukaryotic initiation factor, can initiate protein translation in a cap‐independent manner when a single m6A is present in the 5′‐UTR. IGF2BPs protect mRNA transcripts from degradation under stress conditions and facilitate selective splicing of mRNA. HNRNPA2B1 regulates mRNA splicing and miRNA maturation.

The YTH family proteins are the primary major ‘readers’ that directly bind to m6A sites on RNA. These proteins, containing approximately 100–150 amino acid residues, are present in various eukaryotes and are identified by 14 conserved residues within an α‐helix/β‐sheet structure.^39^, ^40^, ^41^ The three‐dimensional structures and YTH domains of the five types of YTH proteins are shown in Figure 1. Initially identified through RNA affinity chromatography followed by mass spectrometry,^40^ the first YTH protein, YT521‐B, was discovered by Imai et al.^42^ in 1998 during yeast two‐hybrid assays screening for binding partners of the splicing factor TRA‐2β. The gene encoding YT521‐B spans approximately 3200 base pairs (bp) and codes for 712 amino acids. The significance of nuclear YTH protein YT521 in hybrid screenings with splicing factors was further emphasised in subsequent studies.^42^, ^43^ The human splicing factor YT521‐B and its homologs in fruit flies and yeast have a unique 100–150 residue domain that was characterised by Stoilov et al.^44^ in 2002. This domain is known as the YTH domain, which is highly conserved among YT521‐B homologs and primarily avoids protein–protein interactions. Substantial experimental evidence has confirmed that the YTH domain predominantly eschews protein–protein interaction,^43^ establishing it as a newly recognised RNA‐binding domain. The specific mechanism by which the YTH domain recognises m6A remained unclear until 2014 when the structure of the first human YTH complex, YTHDC1‐GG (m6A) CU, was elucidated.^45^ Since then, several structures of protein complexes containing human YTH domains have been reported, including YTHDF1 and YTHDF2 with their m6A‐modified RNA ligands, and the isolated YTH domain of YTHDF2.^39^, ^41^, ^46^, ^47^ Using nuclear magnetic resonance spectroscopy techniques and X‐ray crystallography, the structures in solution and crystalline states of YTH domains from various family members, associated with m6A‐modified RNA oligonucleotides were determined.^39^, ^48^ These YTH domains often have a mixed α‐helix/β‐fold structure, with the β‐fold forming α‐helices around a core globular β‐barrel.^45^ The conserved aromatic pocket within these structures features several highly conserved aromatic residues.^41^ A distinct conserved aromatic cage has been detected in every YTH domain, enabling the recognition of RNA modified with N6‐methyladenosine.^39^, ^41^, ^45^, ^46^, ^47^, ^48^ Ultimately, the YTH domain was revealed to function as a module that recognises m6A in a methylation‐dependent manner.^15^, ^40^, ^45^ The first ‘reader’ discovered was YTHDF2, whose N‐terminal domain promoted mRNA decay while its C‐terminal YTH domain was able to bind mRNA that had been tagged with m6A.^15^ In the cytoplasm, YTHDF1 enhances the translation of m6A‐tagged mRNA,^49^ while YTHDF3 collaborates with YTHDF1 to augment mRNA translation and accelerate the degradation of m6A‐labelled transcripts, interacting with YTHDF2.^50^ YTHDC1, primarily located in the nucleus, regulates selective splicing, nuclear export and gene expression suppression by accelerating the decay of m6A‐tagged transcripts.^51^, ^52^ YTHDC2, the largest m6A‐binding protein in the YTH family at approximately 160 kDa, possesses domains such as YTH, R3H, helicase and DUF1065. Its ATP‐dependent RNA helicase activity plays crucial roles in both mRNA translation and decay.^53^, ^54^ These diverse functions are depicted in Figure 2.

The YTH protein family modulates RNA metabolism by recognising and binding to m6A modifications, influencing transcription, splicing, translation and degradation to affect gene expression and cellular functions. Adipogenesis, haematopoiesis, cancer, immunological responses, viral replication, nervous system development and other physiological and pathological processes are among the physiological and pathological processes that m6A readers are involved in beyond RNA metabolism. For instance, Shi et al.^55^ demonstrated that YTHDF1 is involved in synapse formation and plasticity, thereby influencing learning and memory through the enhanced translation of specific mRNAs in neurons. Similarly, Li et al.^56^ reported that YTHDF2 affects neuronal growth and differentiation by modulating mRNA stability and degradation in neurons. Additionally, YTH proteins have been involved in the pathological processes of neurological disorders, including Alzheimer's disease.^57^, ^58^ Liu's research group found that expression levels of FTO, ELAVL1 and YTHDF2 are altered in Alzheimer's disease, correlating with pathological development and cognitive function.^57^ The YTH protein family is essential in the haematopoietic system, regulating the metabolism of m6A‐modified RNA, influencing the production, differentiation and functionality of blood cells. YTHDF2 is pivotal in maintaining the self‐renewal and multipotency of haematopoietic stem cells (HSCs) by degrading specific mRNAs to prevent excessive differentiation.^59^, ^60^ Furthermore, aberrant YTH protein expression is associated with the advancement of haematological disorders. In acute myeloid leukaemia, for example, YTHDF1 and YTHDF2 are abundantly expressed, facilitating the growth and survival of leukaemia cells.^61^, ^62^ Continued research on YTH proteins could yield novel therapeutic strategies for blood disorders. In the metabolic system, substantial evidence suggests that YTH proteins significantly influence adipogenesis. Huang et al.^63^ found that YTHDF2 inhibits obesity and fat formation by recognising and degrading cyclin A2 and cyclin‐dependent kinase 2 with high m6A levels, thereby suppressing the clonal expansion of preadipocytes through mitosis. Wang et al.^64^ revealed that the deletion of YTHDF2 accelerates autophagy and adipogenesis by stabilising ATG5 and ATG7, offering fresh perspectives on the involvement of YTHDF2 in the development of adipocytes. In the immune system, YTH proteins regulate the generation, differentiation and function of immune cells through their interaction with m6A‐modified RNA. Wang et al. demonstrated the physiological role of m6A modifications in promoting dendritic cell (DC) activation and T‐cell responses by identifying a novel role of METTL3 and YTHDF1‐mediated m6A modification in enhancing the translation of immune transcripts like CD40, CD80 and the TLR4 signalling adapter TIRAP.^65^ In diseases of the immune system, such as autoimmune diseases and chronic inflammation, abnormal expression of YTH proteins can lead to dysregulated immune responses.^66^, ^67^, ^68^, ^69^ In cancer research, the involvement of YTH proteins has garnered significant attention, with numerous studies indicating that YTHDF1 shows elevated expression levels across multiple tumour types, enhancing oncogene expression and cancer development by up‐regulating the translation of m6A‐modified mRNAs in cancer cells.^70^ It has been shown that YTHDF2 regulates the proliferation and metastasis of cancer cells by accelerating the degradation of m6A‐modified mRNAs.^71^, ^72^, ^73^ Additionally, the functions of YTHDC1 and YTHDC2 in tumours are complex, but they also affect the biological activity of tumour cells through the regulation of m6A‐modified RNA.^74^, ^75^, ^76^, ^77^ The YTH protein family also plays significant roles in the cardiovascular system, where YTHDF1 regulates heart function by modulating the translation of key genes in cardiomyocytes,^78^, ^79^, ^80^ and YTHDF2 impacts cardiomyocyte growth and remodelling through its mRNA target transcripts.^81^ Ultimately, the YTH protein family finely regulates RNA modifications, influencing physiological functions across multiple systems, maintaining homeostasis and balance in the body and participating in the pathological processes of various diseases.

## 3 EXPRESSION AND REGULATION OF THE YTH DOMAIN‐CONTAINING PROTEIN FAMILY IN CANCER

**FIGURE 3:**
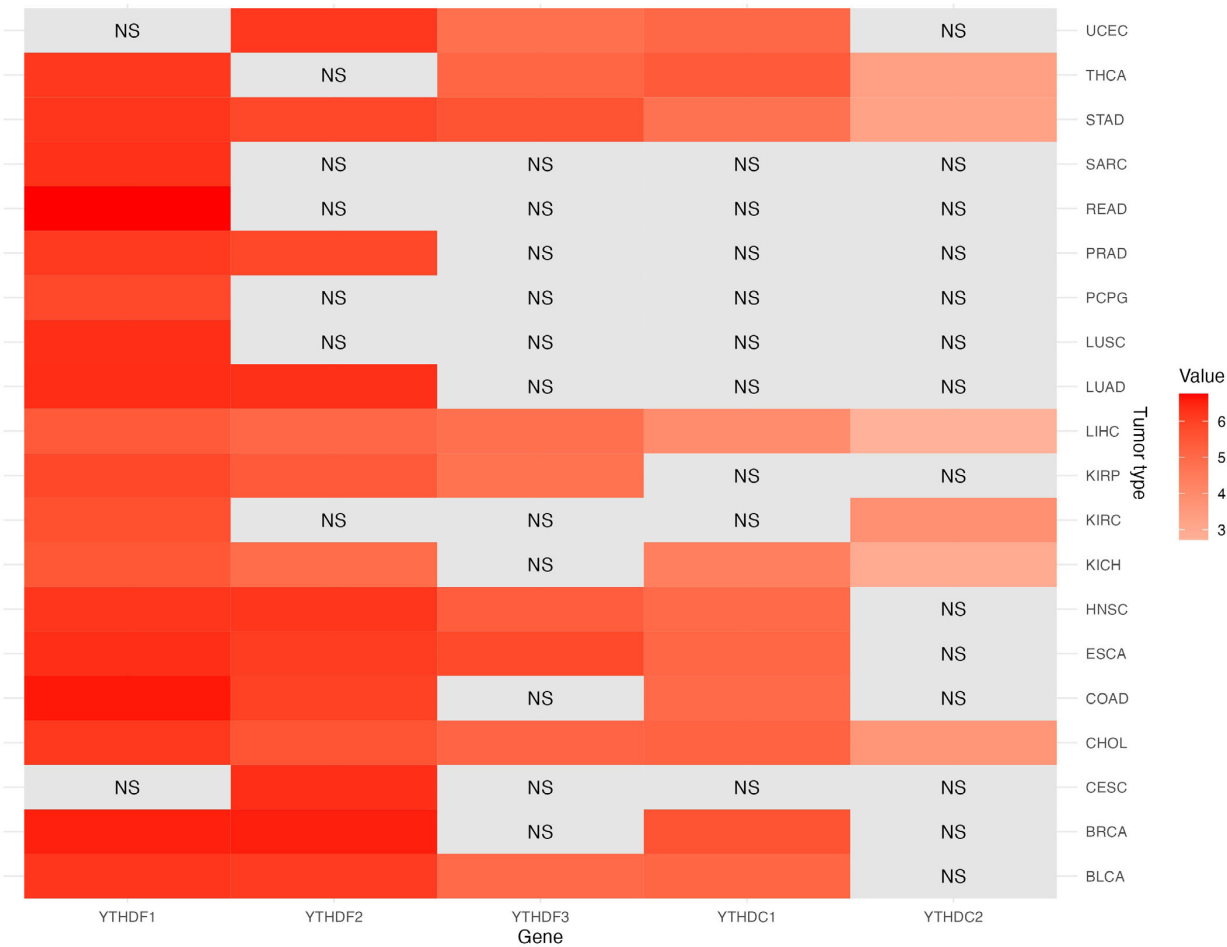
Heatmap of gene expression of YTH domain‐containing proteins. The expression status of YTH domain‐containing proteins in different tumour types was visualised by a heatmap. Red indicates differential gene expression in tumours, from light to deep indicates from low to high expression. ‘Value’ represents the expression level of the gene in tumour tissue relative to normal tissue. The grey area with ‘NS’ indicates that genes containing the YTH domain protein show no significant difference in expression between normal and tumour tissues (*p* > .05).

Accumulating evidence indicates that YTH proteins, as readers of m6A modifications, play a pivotal role in cancer development. Using R for heatmap analysis, the gene expression patterns of the YTH protein family across diverse cancers were examined, revealing significant differences between tumour and normal tissues (Figure 3). Notably, YTHDF1 exhibits high expression levels in colorectal adenocarcinoma, lung adenocarcinoma (LUAD) and colorectal adenocarcinoma tissues, while YTHDF2 is notably up‐regulated in breast cancer (BRCA), head and neck squamous cell carcinoma (HNSC), LUAD and prostate cancer (PRAD). These findings align with previous research. For instance, Zhang et al.^82^ observed significantly elevated YTHDF1 expression in LUAD tissues and demonstrated that LINC00337 promotes LUAD progression by targeting miR‐1285‐3p to regulate YTHDF1 expression. Similarly, Ma's team^83^ elucidated YTHDF1's role in m6A‐induced glycolysis and LUAD tumourigenesis. According to research by Xu's group,^84^ decreasing YTHDF2 increases LATS1's ability to limit tumour growth while high YTHDF2 expression in BRCA cells encourages the creation of tumours. Zhang's team^85^ elucidated YTHDF2's expression pattern in LUAD, showing that KIAA1429, an RNA methyltransferase gene, controls the tumour suppressor gene BTG2's mRNA in a way that is dependent on m6A and YTHDF2, thereby promoting LUAD progression. The differential expression and mechanistic roles of the YTH protein family in cancer have become a focal point of research, given their potential associations with tumour progression, prognosis and therapeutic responses. Further investigation into the functions and mechanisms of YTH proteins in cancer is essential for developing novel therapeutic approaches and targeted treatments.

Numerous studies have identified various factors and mechanisms influencing the YTH protein family. This study conducts an in‐depth exploration of the regulatory mechanisms acting on the YTH protein family at both the transcriptional and post‐transcriptional levels (Figure S1), investigating their impact on cancer development and other diseases. At the transcriptional level, it remains under investigation whether the YTH protein family functions as direct transcription factors or interacts with specific transcription factors. To gain insights into these mechanisms, we performed bioinformatics analysis of YTH family genes was performed using the ENSEMBL database (http://www.ensembl.org/) and the PROMO website (http://alggen.lsi.upc.es/). This analysis enabled the prediction and identification of essential regulatory elements and potential transcription factor‐binding sites (Figure S2 and Table 1). Investigation into the upstream region of the YTHDF1 gene suggests an interaction between the oncogenic transcription factor c‐Myc and YTHDF1. Knockout experiments targeting c‐Myc have demonstrated the suppression of YTHDF1 expression.^86^ Furthermore, studies have shown that HIF‐1α can bind to the promoter region and cause H19 to overexpress. Consequently, H19 plays a regulatory role in gastric cancer (GC) angiogenesis through the YTHDF1/SCARB1 axis,^87^ offering additional insights into the mechanism underlying HIF‐1α/H19/YTHDF1/SCARB1 regulation in GC angiogenesis. Li and colleagues’^88^ research indicates that YTHDF1 promotes the translation of the transcription factor TCF7, leading to increased proliferation of GC cells and inhibition of apoptosis. This finding may provide a novel therapeutic target for GC.^88^

A thorough examination of the expression regulation of YTH proteins at the post‐transcriptional level in RNA maturation, translation and protein processing reveals their significant role in cancer progression. Numerous studies indicate that the YTH protein family, targeted by pivotal miRNAs, regulates oncogenes and tumour suppressor genes. Luo et al.^89^ confirmed YTHDF1's involvement in BRCA progression and its immune microenvironment. They found that miR‐378g inhibits YTHDF1 expression, affecting MDA‐MB‐231 cell proliferation, suggesting YTHDF1 could be a biomarker for poor prognosis and a prospective target for immunotherapy.^89^ Chi et al.^90^ identified a correlation between the hsa–miR‐139‐5p/YTHDF1 axis and hepatocellular cancer (HCC) prognosis, with circMAP2K4 promoting HCC proliferation by sponging hsa–miR‐139‐5p. Zhou et al.^91^ reported that endothelial cell‐mediated extracellular vesicles suppress YTHDF1 expression and activate the Wnt/β‐catenin pathway via miR‐376c, inhibiting non‐small cell lung cancer (NSCLC) malignancy. YTHDF1 is actively engaged in the ceRNA networks associated with esophageal squamous cell carcinoma (ESCC), featuring in interactions such as PAXIP1–AS1 with hsa–miR‐376c‐3p and YTHDF1, THUMPD3‐AS1 linked to hsa–miR‐655‐3p and YTHDF1, and SNHG20 in conjunction with hsa–miR‐655‐3p and YTHDF1.^92^ In glioblastoma (GBM), miR‐346 targets YTHDF1 3’‐UTR, reducing YTHDF1 mRNA levels and suppressing tumour growth.^93^ YTHDF2, identified as a downstream target of miR‐145, plays a role in epithelial ovarian cancer progression by modulating m6A levels.^94^ MiR‐493‐3p directly targets YTHDF2, reducing its expression and suppressing prostatic carcinoma cell proliferation.^95^ According to Chen et al.,^96^ PRAD progression is promoted by activating the KDM5A/miRNA‐495/YTHDF2/m6A‐MOB3B axis. KDM5A binds to the miR‐495 promoter, preventing its transcription. The m6A alteration of MOB3B mRNA is recognised by YTHDF2, which then triggers degradation to restrict MOB3B production. In colorectal cancer (CRC), miR‐6125 down‐regulates YTHDF2, enhancing the stability of m6A‐modified glycogen synthase kinase 3β (GSK3β) mRNA and inhibiting CRC proliferation.^97^ Zhao's^98^ research revealed that circARs down‐regulate YTHDF2 via the A20/NF‐κB axis, promoting systemic lupus erythematosus (SLE). Ma's team^99^ showed that in HCC, miR‐186‐5p up‐regulates FSTL5 by down‐regulating METTL3, inhibiting HCC cell proliferation, migration and invasion. Further investigations indicate that m6A modification‐mediated stability of FSTL5 is mediated by YTHDF2. For HCC, the miR‐186‐5p/METTL3/YTHDF2/FSTL5 axis may provide new treatment avenues.^99^ KDM5B demethylates H3K4me3 on the miR‐448 promoter, inhibiting miR‐448 expression and targeting YTHDF3 and integrin subunit α6, promoting HCC malignancy.^100^ According to Huang et al.,^101^ lincRNA‐Dubr binds to the m6A motif of the YTHDF1/3 complex, facilitating the translation of Tau and calmodulin and preserving axon extension and neuronal migration. Yu et al.^71^ analysed PRMT6 and YTHDF2 expression in GBM using bioinformatics, finding both strongly expressed and correlated with unfavourable outcomes. PRMT6 and CDK9 collaboratively influence the expression of YTHDF2, which in turn attaches to and accelerates the breakdown of antigen‐presenting cell (APC) and GSK3β, negative regulators of the WNT‐β‐catenin signalling cascade, thereby enhancing GBM migration, invasion and epithelial–mesenchymal transition (EMT), implying its suitability as a treatment target for GBM.^71^ According to Li's group,^77^ WTAP promotes ATG5 mRNA translation in liver cancer ferroptosis by up‐regulating ATG5 post‐transcriptionally in a manner that is m6A–YTHDC2 dependent. Inhibiting WTAP or YTHDC2 effectively suppresses ferroptosis and liver cancer progression.

The YTH protein family plays a crucial role in recognising and binding m6A‐modified RNA, influencing the functionality of YTH proteins and other proteins such as METTL3, METTL14, METTL16, FTO and ALKBH5. The YTHDF2‐dependent pathway mediates SOCS2 mRNA degradation. METTL3 suppresses SOCS2 expression in HCC via a m6A–YTHDF2‐dependent mechanism, offering insights into epigenetic changes associated with liver cancer onset.^102^ Wang et al.^103^ discovered that METTL3/YTHDF coupling down‐regulates APC expression, leading to the up‐regulation of the Wnt/β‐catenin pathway in ESCC. Zhou's group^104^ showed that METTL3 enhances the stability of lncRNA LINC00894 mRNA via an m6A–YTHDC2‐dependent pathway, accelerating thyroid carcinoma (PTC) progression and lymph node metastasis through the Hippo signalling pathway. Yang et al.^74^ revealed that KAP1, once activated transcriptionally by MYCN, collaborates with YTHDC1 and METTL3 to form a complex, maintaining MYCN mRNA stability and promoting neuroblastoma oncogenesis in an m6A‐dependent manner. The METTL3 small molecule inhibitor STM2457 targets m6A modification, down‐regulates MYCN expression and inhibits tumour proliferation, providing a new therapeutic strategy for MYCN‐amplified neuroblastoma.^74^ Li and colleagues^105^ demonstrated the pivotal involvement of METTL16 in curtailing PTC progression, achieved by jointly activating SCD1‐mediated lipid metabolism alongside YTHDC2. In NSCLC, ALKBH5 inhibits miR‐107/LATS2‐mediated YAP activity and decreases YTHDF‐mediated YAP production, which in turn suppresses tumour growth.^106^ ALKBH5‐mediated m6A demethylation enhances the mRNA stability of YTHDF1, enabling it to increase YAP translation.^79^ The YTH domain protein family contains intrinsically disordered regions that facilitate liquid‒liquid phase separation,^107^ which occurs on mRNA rather than protein and is enhanced by m6A modifications of YTH proteins in vitro.^108^ Although its expression is noticeably lower in CRC tissues, Zhang and colleagues' research team showed that potassium channel tetramerisation domain containing 15 (KCTD15) functions as an anti‐proliferative agent in CRC cells. Their investigation revealed that FTO/YTHDF2‐mediated m6A alteration controls the mRNA stability of KCTD15. Additionally, by up‐regulating the expression of p53, KCTD15 inhibits the proliferation of CRC cells and triggers apoptosis.^109^ Qiao and colleagues^73^ discovered that inhibiting FTO significantly induces ferroptosis in CRC and enhances CRC cell responsiveness to ferroptosis inducers like erastin and RSL3. At the mechanistic level, elevated FTO levels boost SLC7A11 or GPX4 in an m6A–YTHDF2‐dependent manner, counteracting ferroptotic stress.^73^ Histone lactylation promotes YTHDF2 expression, enabling it to recognise and degrade m6A‐modified PER1 and TP53 mRNA, thereby accelerating the tumourigenesis of ocular melanoma.^110^ HSP90β inhibits STUB1‐induced ubiquitination and degradation of YTHDF2, enhancing growth and the resistance to sorafenib in HCC cells.^111^ Ubiquitination analysis suggests that PFKL up‐regulates the expression of YTHDF3 protein by suppressing EFTUD2‐mediated YTHDF3 ubiquitination.^112^ The solubility and distribution of YTHDC1 in YT bodies are impacted by its phosphorylation by the src kinases c‐src and p59fyn, which causes it to diffuse into the nucleoplasm and interfere with the dynamic interactions of pre‐mRNA protein complexes.^43^, ^113^ A YY1/HDAC2/YTHDC1/ANXA1 axis has been identified that modulates the progression and chemosensitivity of ccRCC.^114^ The comprehension of the YTH domain protein family's regulatory expression will continue to expand and deepen as research advances. The YTH protein family may provide essential foundations and leads for mechanistic studies of tumours and potential therapeutic targets.

## 4 THE ROLE AND MECHANISMS OF THE YTH DOMAIN‐CONTAINING PROTEIN FAMILY IN IMMUNE CELLS

Within the intricate physiological network of the human body, immune cells are crucial. They participate not only in developmental, pregnancy and tissue repair processes but also in pathological scenarios, such as tumours, infections and autoimmune diseases. Recently, the effect of the YTH domain‐containing protein family on tumour immune cells has garnered significant attention, positioning these proteins at the forefront of tumour immunology research. YTH proteins exhibit dual roles, both promoting and inhibiting tumour cells within the TIME, employing multiple mechanisms to evade immune surveillance. The functions and actions of YTH proteins within the TIME are multifaceted and complex. Investigating their interactions with tumour immune cells (Table 1 and Figure S2) promises to offer new opportunities for advancing cancer immunotherapy.

**TABLE 1:** Role of the YTH domain‐containing proteins family in immune cells.

Immune cell	Molecule	signal channel	Role in cell	References
Haematopoietic stem cells	YTHDF2	MYC, CCND1 and AXIN2	Inhibit proliferation of functional HSC	^60^
		Tal1 mRNA	Inhibit the number of functional HSC	^59^
		Chronic pro‐inflammatory pathway	Promote HSC reconstitution of multilineage haematopoiesis	^120^
	YTHDF3	Foxm1, Asxl1	Required for HSC maintenance under stress	^121^
Macrophages	YTHDF1	HMGB1/RAGE, JAK2/STAT3, (P)JAK2/(P)STAT3	Improve the immunoparalysis of macrophages, Th 1/Th 17 and CTL	^127^
		SPRED2, NF‐kB, STAT3	Promote increased tumour infiltration by M1/M2‐like tumour‐associated macrophages and regulatory T cells	^128^
		SOCS3, JAK2/STAT3	Inhibit inflammatory cytokines	^129^
		SOCS1	Offset the excessive and persistent inflammation in the septic response	^126^
	YTHDF2	MAP2K4, MAP4K4, MAPK, NF‐κB	Promote the expression of TNF‐α, IL‐6, IL‐12 and IL‐1β in macrophages	^130^
		RBM4, STAT1	Regulate M1 macrophages polarise	^131^
Dendritic cells	YTHDF1	Lysosomal proteases	Inhibit cross‐presentation of tumour antigens and antigen‐specific CD8 + T cells in vivo	^35^
	YTHDF2	lnc‐Dpf3	Promote DC migration and exacerbate the inflammatory response	^132^
Natural killer cells	YTHDF2	Perforin, granzyme B, IFN‐γ	Regulate melanoma metastasis	^36^
		STAT5	Regulate NK cell survival, proliferation and effector functions	^36^
		Tardbp	Regulate NK cells proliferation	^36^
T lymphocytes	YTHDF2	circRNA	Inhibit innate immunity	^31^
B lymphocyte	YTHDF2	IL‐7	Regulate B cells early development and proliferation o	^136^

### 4.1 Haematopoietic stem cells

Multipotent HSCs can be found in the bone marrow and blood. They have the capacity for self‐renewal, robust differentiation and regeneration, serving as the origin of all blood and immune cells. Common myeloid progenitors (CMPs) and common lymphoid progenitors (CLPs) are two types of multipotent progenitors produced by HSCs. CMPs develop into a variety of cells such as neutrophils, macrophages, eosinophils, basophils, red blood cells and monocytes. In the meantime, CLPs develop into natural killer (NK) cells, T lymphocytes and B lymphocytes.^115^

According to recent research, m6A alteration has a major impact on HSCs differentiation,^116^ shedding light on how m6A governs cell fate in both normal and malignant haematopoiesis.^117^ Targeting this pathway has established as an innovative method for cancer therapy. The absence of YTHDF2 increases the number of transplantable HSCs, while loss of METTL3 impairs differentiation capacity and leads to the accumulation of self‐renewal.^59^, ^118^, ^119^ Haematopoietic‐specific YTHDF2 knockout mice, created using Cre/LoxP systems, exhibit a significant increase in functional HSCs capable of normal lineage differentiation when YTHDF2 is absent. This effect arises from activating Wnt downstream targets, including MYC, CCND1 and AXIN2. YTHDF2 loss prevents the degradation of Wnt target mRNAs and genes associated with survival throughout haematopoietic stress, enhancing gene expression.^60^ Li et al.^59^ demonstrated that YTHDF2 recognises and degrades m6A‐modified mRNAs, such as Tal1 mRNA, essential for stem cell self‐renewal. Conditional deletion of YTHDF2 in mice increases functional HSCs without affecting lineage differentiation or causing haematopoietic malignancies.^59^ Another study indicated that YTHDF2 deficiency chronically activates proinflammatory pathways, leading to progressive bone marrow skewing, diminished lymphoid capacity, expansion of HSCs and the inability of aged YTHDF2‐deficient HSCs to recover multilineage haematopoiesis.^120^ Dang and colleagues^121^ presented evidence of YTHDF3's involvement in haematopoiesis, showing its necessity for maintaining HSCs under stress conditions. YTHDF3 enhances the translation of Foxm1 and Asxl1, crucial regulatory factors for HSC maintenance within HSPCs.^121^ Zhao et al.^122^ reviewed how m6A modification influences HSC formation, self‐regeneration and lineage specialisation, influencing the developmental of HSCs into T and B lymphocytes through various mechanisms. The interaction between the anti‐tumour function of immune cells and tumour cells’ immune evasion mechanisms significantly impacts cancer development and prognosis. It appears that M6A methylation is a promising target for improving immunological therapy for cancer and reducing inflammatory illnesses and infections associated with immune cells. Ongoing research on small molecule m6A drugs aims to boost HSC self‐renewal and generate ample HSCs ex vivo for stem cell transplantation.

### 4.2 Macrophages

Macrophages serve an essential function as APCs within the immune system, acting as sentinels that capture self or foreign antigens and bridging innate and adaptive immunity.^123^, ^124^ Depending on the conditions, macrophages polarise into either M1 or M2 phenotypes. M1 macrophages exhibit anti‐tumour activity, whereas M2 macrophages suppress inflammation, enhance blood vessel formation, facilitate tissue restoration and support tumour metastasis.^125^

Macrophages lacking in METTL14 or YTHDF1 cause septic mice models via CLP or lipopolysaccharide (LPS), which results in increased levels of proinflammatory cytokines and chemokines.^126^ Additionally, YTHDF1 deficiency in macrophages enhances immune responses, Th1/Th17 cell activity and CTL immune function while reducing endothelial injury in septic rats by inhibiting the HMGB1/RAGE pathway, JAK2/STAT3 m6A RNA methylation and (P)JAK2/(P)STAT3 protein expression.^127^ Yin and colleagues^128^ determined that METTL3 deficiency impairs SPRED2 translation mediated by YTHDF1, activating NF‐κB and STAT3 signalling, which boosts tumour‐associated M1/M2‐like macrophages and regulatory T‐cell infiltration. Treponema pallidum infection significantly increases YTHDF1 translation in macrophages, thereby promoting the translation of m6A‐methylated SOCS3 mRNA and controlling anti‐inflammatory responses via the JAK2/STAT3 pathway.^129^ Du and colleagues showed that YTHDF1 counteracts sustained inflammation in sepsis by promoting SOCS1 expression, a negative regulator of macrophage‐mediated inflammation.^126^ In CLP and LPS‐induced septic mouse models, macrophages lacking METTL14 or YTHDF1 maintain higher levels of proinflammatory cytokines and chemokines. Under LPS stimulation, YTHDF2‐deficient macrophages up‐regulate the expression and stability of MAP2K4 and MAP4K4 mRNA, activating MAPK and NF‐κB signalling routes, leading to the increased expression of TNF‐α, IL‐6, IL‐12 and IL‐1β.^130^ Moreover, RNA‐binding motif 4 (RBM4) engages with YTHDF2, targeting STAT1‐mediated glycolysis to polarise M1 macrophages.^131^

### 4.3 Dendritic cells

Initiating T‐cell activation and presenting antigens are two of the immune system's primary functions of DCs. The immune responses mediated by DCs are crucial in the pathogenesis of various tumours. The YTH protein family, widely expressed in DCs, is regulated by external stimuli such as bacterial infection or immune stimulation. Functions of the YTH protein family in DCs include regulating mRNA stability, posttranscriptional modifications and translation, thereby impacting the presentation of antigens and the activation of T‐cells.

Han and their team^35^ determined that m6A methylation via YTHDF1 regulates persistent neoantigen‐specific immunity, contributing to tumour immune evasion. In traditional DCs, YTHDF1 deletion improves in vivo cross‐presentation of tumour antigens and cross‐priming of CD8+ T lymphocytes. By binding to lysosomal protease‐encoding transcripts with m6A marking, YTHDF1 improves the translation of these transcripts in DCs while preventing the cross‐presentation of endogenous tumour antigens and CD8+ T‐cell anti‐tumour responses.^35^ Additionally, YTHDF2 binds to transcripts with specific m6A modifications, affecting the antigen presentation ability of DCs. Hypoxia‐induced adaptation in DCs promotes rapid proliferation and efficient migration to lymph nodes, activating cell‐mediated immune responses. However, CC‐chemokine receptor 7 (CCR7)‐induced lncRNA lnc‐Dpf3 binds directly to HIF‐1α and suppresses LDHA transcription, limiting glycolytic metabolism and migratory capacity. YTHDF2 recognises and accelerates the degradation of m6A‐modified lnc‐Dpf3, promoting DC migration, exacerbating inflammation and disrupting immune homeostasis.^132^

### 4.4 NK cells

By secreting different cytokines and chemokines, NK cells, a subset of innate immune lymphocytes, improve the activities of both the innate and adaptive immune systems.^133^ They are crucial in mediating the host's response against virus‐infected and cancer cells. Ma's study^36^ discovered that YTHDF2 absence in NK cells significantly impairs their responses against tumours and viruses.^36^ YTHDF2 enhances the secretion of perforin, granzyme B and IFN‐γ, thereby regulating melanoma metastasis within the TIME. During viral infections, YTHDF2 regulates perforin production, enhancing NK cell‐mediated antiviral activity. Additionally, YTHDF2 is essential for maintaining NK cell homeostasis, maturation and IL‐15‐mediated survival. A reinforcing feedback cycle between STAT5 and YTHDF2 governs NK cell survival, proliferation and effector functions. Tardbp is a binding target for YTHDF2 that helps NK cells survive and proliferate. YTHDF2 modulates Tardbp mRNA stability, regulating NK cell proliferation and expression.^36^

### 4.5 T lymphocytes and B lymphocytes

T and B lymphocytes are pivotal in adaptive immune responses. Research on m6A modification in lymphocyte development and tumour initiation is still emerging. In tumour immunity, YTHDF1‐deficient mice show enhanced T‐cell responses, resulting in a stronger anti‐tumour CD8+ T‐cell response.^35^ Wang's team,^134^ using single‐cell RNA sequencing and flow cytometry, found that YTHDF1 expression in non‐alcoholic steatohepatitis‐associated HCC (NASH–HCC) cells induces IL‐6 secretion. This mediates the recruitment and activation of myeloid‐derived suppressor cells (MDSCs), leading to CD8+ T‐cell dysfunction and immune suppression in NASH–HCC.^134^ Bao and colleagues^135^ observed in multiple CRC patient cohorts that high YTHDF1 expression negatively correlates with interferon‐γ gene characteristics and the penetration of CD8+ T cells. Their experiments showed that knocking out YTHDF1 induces infiltration of CD4+ T cells, CD8+ T cells and NK cells in CRC tumours and humanised CD34+ mouse models, increasing the manifestation of cytotoxic indicators like granzyme B, interferon‐γ and tumour necrosis factor‐α.^135^ This research indicates that YTHDF1‐driven MDSC accumulation suppressive anti‐tumour immune cells, promoting immune evasion in CRC.^135^ Notably, YTHDF2, in association with m6A‐circRNA, can suppress innate immunity, whereas circRNA acts as an adjuvant inducing activation of antigen‐specific T cells, generation of antibodies and enhancement of anti‐tumour immune responses.^31^ Additionally, recent studies have shown that m6A ‘readers’ are involved in the early growth and proliferation of B cells. While YTHDF2‐mediated transcriptional repression is necessary for IL‐7‐induced pre‐B cell proliferation, YTHDF1 and YTHDF2 are not necessary for the transition from big to small pre‐B cells.^136^ These studies emphasise the crucial immunomodulatory roles of YTHDF proteins in T and B cells and their involvement in tumour progression.

## 5 INVOLVEMENT OF THE YTH DOMAIN‐CONTAINING PROTEIN FAMILY IN TUMOUR IMMUNITY

**FIGURE 4:**
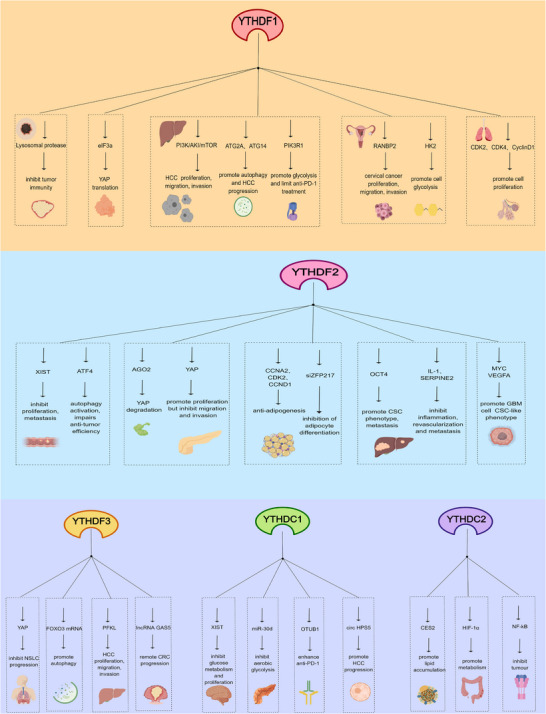
Downstream regulatory molecules and functions of the YTH domain protein family. The YTH domain‐containing protein family plays a crucial role in regulating various signalling molecules. (A) YTHDF1 regulates signalling molecules such as YAP, ATG2A and RANBP2 to participate in the occurrence and development of respiratory, digestive and reproductive system tumours. (b) YTHDF2 regulates signalling molecules such as ATF4, AGO2 and MYC to participate in the occurrence and development of colorectal cancer (CRC), pancreatic cancer and glioblastoma multiforme (GBM). (c) YTHDF3, YTHDC1 and YTHDC2 regulate signalling molecules such as PFKL, lncRNA GAS5 and NF‐KB to participate in the occurrence and development of various tumours, including hepatocellular carcinoma (HCC) and colorectal cancer (CRC). Overall, they play a key role in malignant tumours.

The immune system is crucial in tumour development, growth, invasion and metastasis. While immune cells can attack tumour cells to limit progression, tumours can evade immune detection, facilitating their development and metastasis.^137^ Recent research has increasingly highlighted the role of the YTH domain‐containing protein family in various biological processes, particularly in tumour onset, progression and immune response, as depicted in Figure 4 and Table 2. The YTH protein family significantly impacts tumour and immune regulation by influencing m6A RNA modification. Therefore, a deeper understanding of this family's role in tumour immunity could lead to new therapeutic approaches.

**TABLE 2:** Multiple functions exerted by YTH domain‐containing proteins in various cancers.

Molecule	Cancer type	Target mRNAs	Function	Role in cancer	References
YTHDF1	Melanoma	Lysosomal protease	Translation	Inhibit tumour immunity	^35^
	Colon carcinoma	Lysosomal protease	Translation	Inhibit tumour immunity	^35^
	Breast cancer	ceRNA	Translation	Regulate breast cancer growth	^89^
	NSCLC	CDK2, CDK4, cyclin D1	Translation	Promote cell proliferation	^138^
		RBMS1	Translation	Promote NSCLC metastasis	^139^
	LUAD	DUSP5	Translation	Promote LUAD EMT process and metastasis	^140^
	HCC	NOTCH1	Translation	Promote stemness of HCC cells and enhances sensitivity to targeted therapies	^70^
		ATG2A, ATG14	Translation	Promote hypoxia‐induced autophagy and HCC progression	^154^
		PIK3R1	Translation	Promote cell aerobic glycolysis and limit anti‐PD‐1 treatment	^159^
	NASH–HCC	EZH2–IL6	Translation	Promote MDSC migration and function in NASH–HCC	^134^
	GC	TCF7	Translation	Promote GC cell proliferation and prevent apoptosis	^88^
		HSPH1	Translation	Promote the proliferation and stem potential of GC cell	^143^
	CRC	p65, CXCL1/CXCL2	Translation	Promote CRC progression	^135^
	Cervical cancer	HK2	Translation	Promote cell glycolysis	^160^
		RANBP2	Translation	Promote cell proliferation, migration and invasion	^142^
	–	JAK1	Translation	Promote the immune suppressive function of TIM cells	^171^
	–	MHC‐I	Degradation	Promote immune evasion and resistance to ICIs	^173^
YTHDF2	GBM	MYC, VEGFA	Stability	Promote GBM cell CSC‐like phenotype	^176^
	Glioma	ALKBH5, ZDHHC3	Stability	Promote immune escape	^175^
	Breast cancer	lnc RNA LINC00115, SETDB1, PLK3	Degradation	Promote phenotype and metastasis	^155^
	Liver cancer	OCT4	Translation	Promote CSC phenotype and metastasis	^145^
	CRC	KCTD15	Stability	Regulate the mRNA stability of KCTD15 to control the proliferation and apoptosis of CRC cells	^109^
		XIST	Degradation	Inhibit cell proliferation and metastasis	^146^
		ATF4	Degradation	Stimulate autophagy activation and impairs anti‐tumour efficiency	^166^
	Pancreatic cancer	YAP	Expression	Promote cell proliferation but inhibit migration and invasion	^150^
	HCC	ETV5	Translation	Promote immune evasion and angiogenesis	^144^
		IL‐11, SERPINE2	Degradation	Inhibit inflammation, revascularisation and metastasis	^156^
		FSTL5	Stability	Promote the proliferation, migration and invasion of HCC cell	^99^
	Prostate cancer	PRSS8	Degradation	Promote prostate cancer cell proliferation	^147^
	Colorectal cancer	IFN‐γ–STAT1–IRF1	Stability	Promote sensitivity to PD‐1 blockade	^174^
YTHDF3	CRC	GAS5	Degradation	Promote CRC progression	^151^
YTHDC1	Glioma	XIST	Translation	Inhibit cellular glucose metabolism and proliferation	^164^
	Pancreatic ductal adenocarcinoma	miR‐30d	Translation	Inhibit cell aerobic glycolysis	^163^
	HCC	circHPS5	Cytoplasmic output	Promote HCC tumourigenesis	^148^
	NSCLC	OTUB1	Stability	Promote anti‐PD‐1 efficacy	^37^
YTHDC2	HCC	CES2	Translation	Promote cell lipid accumulation	^167^
YTHDF1/3	NSCLC	YAP	Translation	Inhibit NSLC progression	^152^

### 5.1 YTH domain‐containing protein family promotion of tumour immune evasion and metastasis

Extensive research has shown that m6A readers regulate tumour development, metastasis and drug resistance. In mouse melanoma models, YTHDF1 deficiency restricts lysosomal protease expression in DCs, enhancing cross‐presentation and allowing CD8+ T cells to respond more efficiently to tumour cells. This leads to slower tumour growth and prolonged survival.^35^ Similarly, YTHDF1‐deficient mice in a colon cancer model exhibit suppressed tumour growth and prolonged survival due to the stronger potential of DCs to cross‐present tumour antigens.^35^ In NSCLC studies, the absence of YTHDF1 prevents cell growth and xenograft development by controlling the translation efficiency of CDK2, CDK4 and cyclin D1, suggesting that YTHDF1 loss can inhibit NSCLC progression.^138^ Sun et al. discovered that the RNA‐binding motif single‐stranded‐interacting protein 1 (RBMS1) interacts with YTHDF1 to regulate the translation of S100P via an m6A‐dependent mechanism, facilitating NSCLC metastasis.^139^ First, Fan et al.^140^ demonstrated that DNA hypomethylation and YTHDF1‐mediated abnormal expression of DUSP5 coordinated transfer in LUAD and EGFR–TKI drug resistance by inducing EMTs mediated by TGF‐β/Smad signalling pathways. This suggests that DUSP5 and its derivatives can be utilised for the individualised treatment of patients receiving immunotherapy and targeted therapy for LUAD.^140^ Luo et al.^141^ used bioinformatics databases, in vitro and in vivo detection experiments and data from YTHDF1 knockdown experiments involving RIP‐seq, meRIP‐seq and Ribo‐seq to predict and verify a signalling pathway involving YTHDF1 in HCC. They found that YTHDF1 activates the PI3K/AKT/mTOR signalling cascade, promoting HCC cell proliferation, migration and invasion.^141^ Zhang's team^70^ discovered that YTHDF1 enhances HCC stemness and drug resistance via the YTHDF1–m6A–NOTCH1 epigenetic transcription pathway, predicting HCC recurrence and poor prognosis. This suggests that YTHDF1 antagonists could enhance the sensitivity of HCC molecular targeted therapy.^70^ In cervical cancer cells, YTHDF1 modulation of RANBP2 translation in an m6A‐dependent manner enhances cell growth, migration and invasion, making it a critical target for cervical cancer therapy.^142^ Song et al.^143^ created an in vitro model of gastric mucosal carcinogenesis induced by long‐term low‐dose nitrosamine exposure to investigate YTHDF1's role in gastric epithelial cell carcinogenesis. The study revealed that during MNU‐induced GC, YTHDF1 expression increases, promoting GC cell proliferation and stem potential by regulating the translation of HSPH1. These findings highlight potential therapeutic targets for preventing and treating of environmentally induced GC.^143^

Wen et al.^144^ demonstrate that YTHDF2 inhibits immune escape and blood vessel formation in HCC through the ETV5/PD‐L1/VEGFA axis, identifying it as a potential therapeutic target. Targeting YTHDF2 with an aptamer/liposome containing small interfering RNA effectively inhibits immune escape and blood vessel formation in HCC, causing less harm to normal liver tissue compared to combination therapy. YTHDF2 also promotes the cancer stem cell (CSC) phenotype and metastasis via regulating m6A methylation of OCT4 mRNA and protein expression in liver cancer cells.^145^ In contrast, METTL14 gene knockout in CRC tissues inhibits YTHDF2's recognition of m6A‐methylated X chromosome‐long noncoding RNA (XIST), enhancing XIST expression and promoting CRC progression.^146^ Zhao et al. show that YTHDF2 binds to PRSS8 mRNA in PRAD, promoting its breakdown in a manner dependent on m6A. Practical studies in cell and mouse models indicate that PRSS8 acts as an essential downstream component of the OUT domain–containing ubiquitin aldehyde‐binding protein 1 (OTUB1)–YTHDF2 axis in PRAD.^147^ Rong et al.^148^ observed that YTHDC1 enhances the cytoplasmic export of m6A‐modified circHPS5, which acts as a sponge for miR‐370, accelerating HMGA2 expression and promoting HCC tumourigenesis. Wang's research team substantiates that YTHDC2 exerts tumour‐suppressive effects through the m6A modification‐mediated cylindromatosis/NF‐κB signalling pathway.^149^ Restricting YTH domain‐containing protein family expression can suppress immune evasion and metastasis in malignant tumours, providing novel therapeutic approaches for tumour immunotherapy.

### 5.2 YTH domain‐containing protein family regulates YAP expression in tumour immune regulation

Recent studies have shown that the YTH domain‐containing protein family significantly regulates the expression of Yes‐associated protein (YAP), a core regulatory protein. In pancreatic cancer, YTHDF2 modulates EMT and proliferation differentiation through the HIPPO/YAP axis, aiding tumour immune evasion.^150^ In CRC, m6A‐modified long noncoding RNA transcripts regulate YAP activation, with up‐regulated YTHDF3 recognising and degrading m6A‐containing GAS5, thereby activating YAP signalling and inhibiting CRC progression.^151^ In NSCLC, METTL3 enhances YAP stability and translation efficiency by increasing YAP m6A levels and recruiting YTHDF1/3 and eIF3b.^152^ Furthermore, YAP expression is modulated by YTHDF3 in conjunction with YTHDF1 and YTHDF2, independent of m6A. AGO2 system‐mediated YAP mRNA degradation is facilitated by YTHDF2; YTHDF3 binds to YAP precursor RNA; and YTHDF1 stimulates YAP mRNA translation by interacting with eIF3a.^106^

### 5.3 YTH domain‐containing protein family targeting metabolism to regulate the TIME

Recent studies have demonstrated that the YTH domain‐containing protein family is pivotal in modulating the TIME under hypoxia, metabolic reprogramming, acidity and immune suppression, impacting immune evasion. In hypoxic HCC TIME, down‐regulation of METTL3 reduces YTHDF1 expression, stabilising FOXO3 transcripts and decreasing sensitivity to sorafenib.^153^ Li et al.^154^ showed that in hypoxic conditions across multiple HCC models, HIF‐1α‐driven YTHDF1 expression enhances the translation of autophagy‐related genes ATG2A and ATG14, contributing to HCC progression. Thus, YTHDF1 emerges acting as a promising marker for prognosis and therapy in HCC.^154^ Luo's team discovered that lncRNA LINC00115 enhances SETDB1 methylation of PLK3 at K106 and K200 in drug‐resistant BRCA. This methylation reduces HIF1α phosphorylation by PLK3, stabilising HIF1α, which up‐regulates ALKBH5 and reduces m6A modification of LINC00115, decreasing its degradation by YTHDF2 and increasing LINC00115 stability.^155^ This positive feedback loop promotes the BCSC phenotype, enhancing chemoresistance and metastasis in triple‐negative BRCA. Another study revealed that HIF‐2α‐induced inhibition of YTHDF2 expression promotes the degradation of IL‐11 and SERPINE2 mRNAs, stimulating inflammation, angiogenesis and metastasis in HCC. Treatment with the HIF‐2α antagonist PT2385 restores YTHDF2 expression and inhibits HCC progression.^156^ Tanabe et al.^157^ identified that YTHDC2 may promote colon cancer metastasis by enhancing HIF‐1α translation, highlighting YTHDC2 as a potential diagnostic marker and therapeutic target. Deleting YTHDC2 attenuates Twist1 mRNA translation, a key regulatory factor of HIF‐1α and EMT.^158^

The YTH domain‐containing protein family significantly reprograms the metabolism of major biomolecules, including glycolysis, amino acids and lipids, influencing tumour progression and treatment response. In HCC, circRHBDD1 recruits YTHDF1 to accelerate the translation of PIK3R1 mRNA, promoting aerobic glycolysis and reducing anti‐PD‐1 treatment efficacy.^159^ Targeting the circRHBDD1/YTHDF1/PIK3R1 axis shows immune‐enhancing effects. Wang et al.^160^ demonstrated that METTL3 up‐regulates and stabilises HK2 mRNA through YTHDF1‐mediated m6A regulation, enhancing glycolysis in cervical cancer. In both cervical and liver cancers, m6A modification in the 5′‐UTR of PDK4 stabilises PDK4 via the YTHDF1/eEF‐2 complex and IGF2BP3, promoting glycolysis and cancer progression.^161^ Fu et al.^162^ found that USF1 inhibits glycolysis and the advancement of PRAD via activating ALKBH5 to stabilise FLII mRNA in a YTHDF2‐dependent m6A way. Zhou et al.^112^ identified a reinforcing regulatory cycle between YTHDF3 and phosphofructokinase PFKL in HCC glucose metabolism, with YTHDF3 enhancing PFKL mRNA and protein levels by inhibiting its degradation through m6A modification. Hou et al.^163^ explored the YTHDC1/miR‐30d/RUNX1 pathway in pancreatic ductal adenocarcinoma, showing that YTHDC1 promotes miR‐30d biosynthesis, which targets RUNX1 to regulate SLC2A1 and HK1 expression, thereby inhibiting aerobic glycolysis. Additionally, YTHDC1 predominantly recognises m6A residues on XIST, leading to X‐linked gene silencing and influencing glucose metabolism in glioma cells.^164^ Methionine metabolism has been shown by Li et al.^165^ to enhance immunological checkpoint gene m6A methylation, comprising PD‐L1 and V‐domain Ig suppressor of T‐cell activation, and using YTHDF1 can increase the expression of both. Methionine or YTHDF1 deficiency can inhibit tumour growth and synergise with PD‐1 blockade to improve anti‐tumour immune therapy by restoring CD8+ T‐cell cytotoxicity. In CRC, glutamine enhances m6A methylation and YTHDF2‐mediated degradation of ATF4 mRNA, stimulating autophagy and impairing anti‐tumour efficacy.^166^ Takemoto et al.^167^ showed that YTHDC2 down‐regulates CES2 expression by recognising m6A residues in the 5′‐UTR of CES2, regulating lipid accumulation in HCC cells. During adipogenesis, YTHDF2 recognises and degrades methylated CCNA2 and CDK2 mRNAs, reducing their levels and impairing adipogenesis. Epigallocatechin gallate in green tea increases m6A modification on CCNA2 and CDK2 and enhances YTHDF2 expression,^168^ exerting antiadipogenic effects. YTHDF2 also degrades methylated CCND1 mRNA, down‐regulating CCND1 and suppressing adipogenesis. YTHDF2 knockdown mitigates siZFP217‐induced inhibition of adipocyte differentiation.^169^ Wang's research team^64^ demonstrated that Atg5 and Atg7 are targets of YTHDF2. FTO silencing increases m6A levels on Atg5 and Atg7 transcripts, allowing YTHDF2 to capture these transcripts, reducing their degradation and enhancing protein expression, thereby mitigating autophagy and lipogenesis.^64^ By identifying the m6A modification sites around the FOXO3 mRNA stop codon and enlisting eIF3a and eIF4B to improve FOXO3 translation, YTHDF3 stimulates autophagy.^170^ Xiong et al. revealed that METTL3 mediates lactylation‐driven m6A modification of JAK1 mRNA in tumour‐infiltrating myeloid (TIM) cells, with YTHDF1 enhancing JAK1 translation and subsequent STAT3 phosphorylation, thus enhancing the immunosuppressive function of TIM cells.^171^ These studies underscore the dynamic and reversible regulation of the YTH domain‐containing protein family in the TIME, offering new targets for anti‐tumour immune therapy. Immune therapies targeting metabolism are emerging as effective methods for tumour treatment.

### 5.4 YTH domain‐containing protein family and immune checkpoint blockade immunotherapy

The therapeutic landscape has been profoundly altered by immunotherapy, particularly immune checkpoint blockade (ICB), which has revolutionised the treatment of a number of malignancies. However, numerous patients either exhibit no response to ICB or acquire resistance, limiting its effectiveness. Research has underscored the importance of m6A modification and associated regulatory factors, including the YTH domain protein family, as promising targets for enhancing tumour immunotherapy. The response to PD‐1/PD‐L1 blockade is influenced by various tumour and TIME characteristics.^172^ Han et al.^35^ observed that YTHDF1‐deficient mice undergoing PD‐L1 blockade exhibited more extensive tumour regression than untreated YTHDF1−/− or WT mice subjected to anti‐PD‐L1 therapy, suggesting that YTHDF1 as a potential anti‐tumour immunotherapy target. Li's group discovered that YTHDF1 deletion or a methionine‐restricted diet combined with PD‐1 inhibition can effectively suppress tumour growth by reestablishing CD8+ T cell infiltration.^165^ Wang et al.^134^ discovered that targeting YTHDF1 with siRNA enhances anti‐tumour immunity in NASH–HCC by inhibiting the EZH2‐IL6 pathway, augmenting the efficacy of anti‐PD1 therapy. This identifies YTHDF1 as a novel target for improving anti‐PD‐1 therapy response in NASH–HCC.^134^ Bao et al.^135^ demonstrated that in CRC, YTHDF1 expression recruits immunosuppressive MDSCs by activating the m6A–p65–CXCL1 axis, inhibiting T cell function and promoting CRC progression. Combined targeting of YTHDF1 and anti‐PD‐1 therapy shows promising anti‐tumour effects in CRC, highlighting YTHDF1 as a significant therapeutic target.^135^ Li et al. revealed that intrinsic tumour YTHDF1 facilitates immune evasion and resistance to immune checkpoint inhibitors (ICIs) by promoting MHC‐I degradation. The deletion of YTHDF1 can turn ‘cold tumours’ into responsive ‘hot tumours’, increasing the effectiveness of ICI.^173^ Wang and colleagues^174^ modified the TME and tumour‐infiltrating cells to improve the pMMR–MSI‐L CRC's immunotherapy response. Depleting METTL3 or METTL14 stabilises STAT1 and IRF1 mRNA through YTHDF2, promoting IFN‐γ‐STAT1‐IRF1 signalling,^174^ thereby enhancing sensitivity to PD‐1 blockade. This uncovers a gene methylation mechanism that amplifies the response of pMMR–MSI‐L CRC to PD‐1 inhibition, offering new therapeutic avenues and novel biomarkers. Tang's team^175^ found that in glioma, ALKBH5 regulates the stability of ZDHHC3 mRNA in a YTHDF2‐dependent manner, promoting PD‐L1‐mediated immune escape. This highlights a new role for ALKBH5 and YTHDF2 in glioma, presenting a promising target for glioma immunotherapy.^175^ Another study indicated that YTHDF2 stabilises MYC and VEGFA in GBM stem cells through m6A‐dependent mechanisms, promoting CSC‐like phenotypes in tumour cells.^176^ CSCs, or tumour‐initiating cells, possess the ability to self‐renew and differentiate into multiple lineages, contributing to intratumoural heterogeneity (ITH), tumour progression, recurrence, metastasis and resistance to immunotherapy.^177^, ^178^ Immune privilege is the ability of CSCs to elude innate and adaptive immune monitoring.^179^ CSCs multiply symmetrically, enlist immunosuppressive cells and use additional strategies to avoid immune monitoring during the tumour immune escape (TIE) phase.^180^, ^181^ Dong et al.^182^ demonstrated that the TIMELESS (TIM) gene promotes immune evasion and progression in BRCA by up‐regulating PD‐L1 expression through its interaction with c‐Myc. TIM influences CSC self‐renewal and enhances TIE by regulating PD‐L1 expression,^183^ providing new insights into BRCA treatment. These findings suggest a close relationship between YTHDF2 and CSCs with TIE in GBM, indicating that investigating YTHDF2's role in TIE could develop more effective immunotherapeutic targets for GBM.

Previous studies have demonstrated that combining anti‐angiogenic antibodies with ICIs effectively treats HCC more than monotherapy.^184^, ^185^ Wen's team^144^ found that YTHDF2 promotes immune escape and angiogenesis in HCC by up‐regulating ETV5, inducing the transcription of PD‐L1 and VEGFA. Based on this mechanism, they applied the A/Lipo/si‐YTHDF2 complex to target YTHDF2 in HCC treatment, causing less damage to normal liver tissue compared to previous combination therapy regimens.^144^ In melanoma, FTO knockdown increases m6A methylation of key oncogenes like PD‐1, CXCR4 and SOX10, promoting YTHDF2‐mediated mRNA degradation and making melanoma cells more susceptible to interferon‐γ and anti‐PD‐1 therapy.^186^ Kazuo Tsuchiya's research in NSCLC found that knocking out YTHDF1 and YTHDF2 elevated PD‐L1 expression in tumours and changed several immune‐related genes. Elevated levels of YTHDF1 and YTHDF2 correlate with a good survival outlook, more tumour‐infiltrating lymphocytes and PD‐L1 down‐regulation in NSCLC patients, suggesting their potential as prognostic markers and therapeutic targets related to the TIME in lung cancer.^187^ Wang et al.^188^ demonstrated that after ionising radiation (IR), deleting YTHDF2 in myeloid cells enhances anti‐tumour immune response and counteracts tumour radioresistance through changing MDSC differentiation and preventing their infiltration and function. The small molecule inhibitor DC‐Y13‐27, identified through screening and targeting YTHDF2, can overcome MDSC‐induced immunosuppression and improve the efficacy of IR and/or anti‐PD‐L1 combination therapy, offering a new combined treatment strategy for tumour immunotherapy.^188^ METTL3 facilitates IGF2BP3 circularisation through YTHDC1, triggering PD‐L1 deubiquitination and resulting in CD8+ T‐cell‐mediated immune evasion. Additionally, METTL3 stabilises OTUB1 mRNA in a PKP3‐dependent manner, enhancing the efficacy of anti‐PD‐1 therapy in NSCLC.^37^ YTHDC2 expression and immune scoring and Spearman correlation analysis of YTHDC2 gene expression revealed a significant positive correlation between immune cell infiltration levels, including CD8+ T cells, CD4+ T cells, neutrophils, bone marrow DCs, macrophages and B cells ^38^. Recent studies link low YTHDC2 expression with poor prognosis, apoptosis activation and ubiquitin‐mediated protein degradation in HNSCC.^189^ Li et al.^190^ determined through IHC analysis that YTHDC2 acts as an anticancer gene, exhibiting high levels of expression in normal tissues and reduced expression in tumour tissues. YTHDC2 serves as a prognostic marker for HNSCC and interacts with immune infiltration of HNSCC tumours.^190^ In HNSCC, YTHDC2 expression correlates with the infiltration levels of B cells, CD8+ T cells, CD4+ T cells, neutrophils and DCs, with a positive association with CD4+ T‐cell subpopulations. These studies affirm that targeting YTHDF1/2 and YTHDC1/2 in human cancers can reactivate the anti‐tumour immune response and enhance the efficacy of ICI therapy, offering new avenues for targeted therapeutic approaches. Understanding these mechanisms will facilitate the optimisation and identification of novel immune therapy strategies and their combinations.

## 6 YTH DOMAIN‐CONTAINING PROTEIN FAMILY IS ASSOCIATED WITH OTHER IMMUNE‐RELATED DISEASES

The YTH domain‐containing protein family plays a crucial role in immune‐related diseases through diverse mechanisms (Table 3). These proteins directly regulate immune cell function and finely tune various signalling pathways, shaping broader immune responses. In autoimmune diseases, the abnormal expression or dysregulation of YTH proteins is linked to disease onset and progression. Additionally, YTH proteins are essential in immune responses to specific viral or bacterial infections and may even be associated with allergic reactions. Their involvement in the progression and severity of numerous immune‐related conditions suggests significant potential for therapeutic interventions.

**TABLE 3:** Main functions of YTH domain‐containing proteins in autoimmune disease and infectious diseases.

	Disease	Protein	Main functions	References
Autoimmune disease	SLE	YTHDF2	Reduce the risk of disease occurrence	^66^
	RA	YTHDF2	Associated with blood cells	^193^
	T1DM	YTHDC1	Regulate T1DM mRNA expression	^67^
	DN	YTHDC1	Nosogenesis	^194^
	EAU	YTHDC2	Alleviate EAU and inhibit pathogenic Th17 cell responses	^195^
Infectious diseases	EBV	YTHDF1	Down‐regulate EBV gene expression	^196^
		YTHDF2	Down‐regulate EBV replication	^201^
	HCV	YTHDF protein	Down‐regulate infectious viral particles	^198^
	ZIKV	YTHDF protein	Increase viral replication	^199^
	EV71	YTH protein family	Increase viral genomic	^200^
	CHIKV	YTHDF1	Inhibit CHIKV replication and maturation	^203^
	HIV‐1	YTHDF protein	Inhibit HIV‐1 virus replication	^204^
		YTHDF protein	Increase viral transcription and protein levels	^205^
	KSHV	YTHDF2	Up‐regulate virion production	^16^
		YTHDF2	Antiviral mechanism	^206^
		YTHDC1	Promote KSHV cleavage replication	^207^
	FMDV	YTHDF2	Inhibit viral replication	^212^
Other immune‐related diseases	Asthma	YTHDF3, YTHDC1	Abnormal expression	^213^
	LIRI	YTHDF3	Enhanced hypoxia/reoxygenation‐induced cell injury in human pulmonary bronchial epithelial cells	^214^

SLE, systemic lupus erythematosus; RA, arthritis deformans; T1DM, type 1 diabetes; DN, diabetic nephropathy; EAU, experimental autoimmune uveitis; EBV, Burkitt's lymphoma virus; HCV, hepatitis C virus; ZIKV, zika virus; EV71, enterovirus 71; CHIKV, chikungunya virus; HIV‐1, human immunodeficiency virus type 1; KSHV, Kaposi sarcoma‐associate herpesvirus; FMDV, foot‐and‐mouth disease virus; LIRI, lung ischaemia and reperfusion injury.

### 6.1 YTH domain‐containing protein family and autoimmune diseases

Studies have demonstrated a connection between m6A alteration and SLE, with individuals with SLE displaying down‐regulated METTL3, METTL14, WTAP, FTO, ALKBH5 and YTHDF2 mRNA expression.^66^ Analyses using logistic and comprehensive logistic regression indicate that reduced expression of YTHDF2 or ALKBH5 mRNA could be linked to a higher risk of SLE.^191^, ^192^ Similarly, associations between YTHDF2 mRNA expression and different immune cell counts are revealed by decreased mRNA expression levels of ALKBH5, FTO and YTHDF2 in the peripheral blood mononuclear cells of RA patients.^193^

Recent studies have identified changes in YTH domain protein expression in autoimmune diseases. Significant differences were observed in one ‘writer’ (METTL3) and three ‘readers’ (NRNPA2B1, IGF2BP2 and YTHDC1) between type 1 diabetes (T1DM) patients and healthy subjects, implicating YTHDC1 in regulating mRNA expression in T1DM.^67^ In diabetic nephropathy (DN), METTL3, ADAR1 and DNMT1 were up‐regulated, while YTHDC1 was down‐regulated in DN podocytes compared with normal glucose‐cultured cells. This suggests that m6A methylation regulation and immune infiltration control are crucial in DN pathogenesis.^194^ Zhao and colleagues^195^ found that METTL3 promotes ASH1L mRNA stability and expression in a YTHDC2‐dependent manner, inhibiting the pathogenic Th17 cell response in experimental autoimmune uveitis and its animal model. These findings highlight the critical roles of METTL3 and YTH domain proteins in regulating pathogenic Th17 responses and suggest their potential in treating autoimmune diseases. Further investigation is needed to elucidate their precise molecular and epigenetic mechanisms for improving immune kidney disease treatments.

### 6.2 YTH domain‐containing protein family and infectious diseases

Recent research has demonstrated the significant role of YTH domain‐containing proteins in the host's response to viral infections, acting as either proviral or antiviral factors. YTHDF proteins, for example, exhibit antiviral effects in the lifecycles of Epstein‒Barr virus (EBV),^196^ hepatitis B virus,^197^ hepatitis C virus,^198^ zika virus,^199^ and enterovirus 71.^200^ YTHDF1 accelerates the degradation of m6A‐modified EBV mRNAs by recruiting the RNA degradation complex of ZAP, DDX17 and DCP2.^196^ Down‐regulating EBV gene expression.^196^ During EBV replication, YTHDF2 is cleaved by caspase at the D166 and D367 sites, reducing YTHDF2 expression, increasing caspase‐8 protein levels and enhancing EBV replication. Sugiokto and colleagues^201^ demonstrated that PIAS1 enhances the SUMOylation of YTHDF2 at lysine positions K281, K571 and K572, which aids in the degradation of viral RNA and reduces EBV replication. Additionally, PIAS1 is involved in the SUMOylation of YTHDF1 and YTHDF3, contributing to the restriction of EBV replication.^201^ These results underscore a distinct mechanism through which the YTHDF protein family regulates EBV replication via PIAS1‐mediated SUMOylation, emphasising their critical role in maintaining viral mRNA stability and controlling EBV replication.^202^ Targeting YTHDF1/2 for EBV‐related therapies holds potential. Additionally, YTHDF1 inhibits the replication and maturation of chikungunya virus (CHIKV) by binding to it.^203^ The role of YTHDF proteins in human immunodeficiency virus type 1 (HIV‐1) transcription and replication remains debated. Some studies suggest that YTHDF proteins primarily suppress HIV‐1 replication by decreasing reverse transcription,^204^ contradicting earlier beliefs that they enhance viral transcription and protein levels.^205^ This indicates that YTHDF‐mediated regulation might rely on the specific stages of the viral lifecycle and requires further investigation. The role of m6A modification in the oncogenic human DNA virus Kaposi's sarcoma‐associated herpesvirus (KSHV) is similarly contentious. METTL3 and YTHDF2 have roles in KSHV‐infected B cells, with reduced expression significantly decreasing viral particle production.^16^ However, other studies suggest that m6A and YTHDF2 play antiviral roles during KSHV's lytic replication process.^206^ YTHDC1 facilitates KSHV lytic replication by promoting replication and transcription activator splicing.^207^ These findings imply that m6A modification and related factors, such as YTHDF2 and YTHDC1, could be potential targets for developing new KSHV antiviral therapies.

Deletion of YTHDF2 leads to increased up‐regulation of interferon‐stimulated genes in response to viral infection or inactivated viral agents, inhibiting the replication of various viruses in an interferon signal‐dependent manner.^208^ Another study demonstrated that YTHDF3 functions as an enhancer of the antiviral JAK/STAT axis as a reaction to positive single‐stranded RNA virus infection, promoting IFN‐1‐mediated gene regulation programs in infected cells.^209^ Under steady‐state conditions, YTHDF3 cooperates with PABP1 and eIF4G2 to facilitate FOXO3 mRNA translation, suppressing the expression of interferon‐stimulated genes, which play pivotal roles in the IFN‐dependent antiviral immune response, thereby maintaining the host's antiviral immune function.^210^ Recently, Robert et al.^211^ discovered that α‐herpesvirus‐induced remodelling of the cellular transcriptome leads to the loss of m6A‐modified transcripts, constituting an immune evasion strategy. Their experiments showed that while the instability of m6A‐modified transcripts mediated via YTHDF proteins is not necessary for α‐herpesvirus replication in cells lacking interferon, it paradoxically inhibits the type I interferon response and stimulates viral protein production in primary epithelial cells. Through this mechanism, specific α‐herpesviruses and other viral types modulate the innate immune response through the preferential induction of the degradation of host mRNA containing m6A modifications. Liu's research group proposed a model to elucidate the potential mechanisms of GTPBP4 and YTHDF2 in antiviral innate immune responses and autophagy. They found that the foot‐and‐mouth disease virus (FMDV) structural protein VP1 interacts with YTHDF2 in the AKT–MTOR‐dependent autophagy pathway, leading to YTHDF2 degradation. This results in increased levels of GTPBP4 mRNA and protein, thereby inhibiting FMDV‐induced type I interferon production and promoting virus replication.^212^ In conclusion, multiple studies underscore the vital regulatory role of YTH domain‐containing proteins in the immune response against viral infections.

### 6.3 YTH domain‐containing protein family and asthma and organ transplantation

YTHDF proteins are associated with immune regulation in asthma and organ transplantation. Asthma, a chronic inflammatory respiratory disease, involves complex immune and inflammatory responses. The YTH protein family plays a pivotal role in these processes, potentially impacting asthma development and pathophysiology. Under the U‐BIOPRED (Unbiased Biomarkers Predictive of Respiratory Disease Outcomes) program, Sun et al.^213^ systematically evaluated the impact of m6A modification on immune microenvironment characteristics in patients and healthy controls. They found abnormal expression of YTHDF3 and YTHDC1 in many severe asthma patients, suggesting alterations in the immune microenvironment and influencing eosinophil activity relevant to severe asthma.^213^ These findings, although limited in direct evidence, highlight the critical role of m6A modification in severe asthma and its potential to guide future immune therapy strategies. Lung ischaemia‒reperfusion injury (LIRI), a common consequence of lung transplantation, is related to the pathogenesis of ischaemia‒reperfusion injury, with m6A methylation playing a significant role. Transcriptome analysis of rat lung tissues with LIRI revealed differential expression of YTHDF3 and IGF2BP2. Further research by Xiao and others^214^ showed that knocking down YTHDF3 or IGF2BP2 could mitigate hypoxia/reoxygenation‐induced human bronchial epithelial cell damage by deactivating the P38, AKT, ERK1/2 and NF‐κB pathways. These findings suggest that targeting YTHDF3 and IGF2BP2 could be a promising approach for reducing LIRI in lung transplantation, although further studies are needed to fully explore their therapeutic potential.

## 7 YTH DOMAIN‐CONTAINING PROTEIN FAMILY AS EMERGING THERAPEUTIC DRUGS AND TARGETS

The YTH protein family has been linked to a number of diseases, including immune‐related disorders and cancer, according to recent research. As a result, it is becoming more widely acknowledged that members of the YTH family have the potential to be novel therapeutic agents. Li et al.^215^ utilised various bioinformatics tools and found that the YTH domain family plays a crucial role in m6A modification across different cancers, leading to differential protein expression. They further confirmed the potential of the YTH domain family in predicting cancer prognosis and sensitivity to immunotherapy.^215^ Targeting the YTH domain can enhance the immune system to combat tumours, autoimmune diseases and infectious diseases.

The YTH domain protein family is also involved in the development of tumour drug resistance. Yang et al.^216^ evaluated the expression of 21 m6A‐related genes in 1039 lung cancer patients and 107 controls through comprehensive bioinformatics analysis. They analysed these genes’ relationships with the TME and drug resistance, finding that etoposide sensitivity negatively correlated with ELAVL1, HNRNPC, RBM15B, YTHDF2 and CBLL1. This study revealed that m6A‐related genes significantly contribute to lung cancer, with the expression levels of YTHDF1, YTHDF2, ELAVL1 and ZC3H13 being crucial for predicting and treating lung cancer, providing new therapeutic targets.^216^ Zhang et al.^217^ discovered that METTL3 promotes LDHA expression by stabilising hypoxia‐inducible factor α transcription and initiates LDHA translation through methylation of its CDS region and recruitment of YTHDF1. Targeting the METTL3/YTHDF1/LDHA axis significantly enhances CRC cells’ sensitivity to 5‐FU in both in vivo and in vitro settings, presenting a potential therapeutic target to overcome 5‐FU resistance.^217^ Ou et al.^218^ found that knocking out the GPRC5A gene in triple‐negative BRCA reduces tumour cell metastasis and resistance to docetaxel. The METTL3/YTHDF1 axis up‐regulates GPRC5A expression through m6A methylation, inhibits LAMTOR1 ubiquitination to prevent its degradation and activates the mTORC1/p70S6K signalling pathway by recruiting mTORC1 to lysosomes, promoting docetaxel resistance and liver metastasis.^218^ Yin et al.^219^ identified m6A‐modified RIPK4's role in carcinogenesis and drug resistance. YTHDF1 up‐regulates m6A‐modified RIPK4 by inhibiting its mRNA degradation, promoting NF‐κB phosphorylation, tumourigenesis and cisplatin resistance in ovarian cancer cells.^219^ Yao et al.^220^ demonstrated that the KIAA1429/m6A/YTHDF1 axis promotes chronic myeloid leukaemia (CML) cells’ proliferation and drug resistance by increasing RAB27B mRNA stability and expression. They developed Rucaparib, a novel anticancer drug that inhibits KIAA1429 expression, suppressing CML cell proliferation, promoting apoptosis and increasing sensitivity to imatinib. Animal experiments showed that Rucaparib effectively inhibits tumour growth without significant toxicity in mice, indicating its high potential for practical clinical purposes,^220^ although more research efforts are essential to explore its therapeutic effects alone or combined with other treatments. Liao et al. demonstrated that inhibiting HSP90β enhances liver cancer cells’ sensitivity to sorafenib by inducing the interaction between STUB1 and YTHDF2.^111^ Huang's team found that YTHDF2 is up‐regulated in intrahepatic cholangiocarcinoma (ICC) tissues, particularly in chemoresistant ICC, exhibiting oncogenic and cisplatin‐desensitising effects.^221^ Overexpressed YTHDF2 promotes CDKN1B degradation in an m6A‐dependent manner, enhancing cell proliferation and inhibiting apoptosis, thus reducing ICC cells' sensitivity to cisplatin. Combining YTHDF2 siRNA and cisplatin significantly enhanced cisplatin's anti‐tumour effects in chemoresistant ICC PDX models, suggesting new combined therapeutic strategies for ICC.^221^ Su and colleagues^222^ discovered that low YTHDC1 expression in bladder cancer patients correlates with cisplatin sensitivity, DNA damage and reduced PTEN expression. They identified a novel epitranscriptomic mechanism where YTHDC1 regulates the PTEN/PI3K/AKT signalling pathway in an m6A‐dependent manner, affecting chemotherapeutic outcomes in bladder cancer.^222^ The PI3K/AKT inhibitor MK2206 inhibits YTHDC1 expression, increases cisplatin resistance and activates the DNA damage response.^222^ These results suggest that YTHDC1 is key in influencing cisplatin resistance in bladder cancer. Sun et al.^223^ confirmed that METTL14‐induced m6A mRNA methylation inhibits pri‐miR‐17 mRNA degradation by reducing YTHDC2 recognition of the ‘GGACC’ binding site, regulating mitochondrial function and playing a critical role in CRC resistance to 5‐FU. This study links m6A modification of pri‐miRNA, mitochondrial dynamics and chemoresistance, providing new potential targets for CRC therapy. Chen et al.^224^ reported that high LIM kinase 1 (LIMK1) expression in CRC cells promotes cell proliferation and increases 5‐FU resistance. Down‐regulation of YTHDC2 reduces the recognition and binding of the m6A site ‘GGACA’ in LIMK1 mRNA, increasing its stability and expression.^224^ Overexpressed LIMK1 promotes eIF2α phosphorylation, induces ER stress and facilitates stress granule formation, ultimately leading to 5‐FU resistance. These studies provide new insights into the potential epigenetic mechanisms of the YTH family in tumour resistance and suggest strategies to overcome it. Is tumour resistance more related to YTH family‐mediated m6A modification, or does abnormal tumour immune regulation mediated by the YTH family lead to cancer cell resistance? Further research is required to explore this question.

Researchers have begun exploring the potential of m6A epigenetic mechanisms as novel drug targets, with an initial focus on the FTO enzyme.^225^ Recent studies have identified effective inhibitors of METTL3^226^ and small molecule ligands binding to the METTL3–14–WTAP complex,^227^ with their actions verified through experiments. The YTH domain is another potential target for immunotherapy and pharmacology,^228^, ^229^ although inhibitors specifically targeting YTHDF or YTHDC proteins are limited. Micaelli and colleagues^230^ identified a type I inhibitor of the YTH domain in YTHDF proteins called ebselen. Through high‐throughput screening of small molecules, they delineated its binding interaction with the YTH protein family using X‐ray and NMR structural studies. Ebselen associates with the YTH domain of both YTHDF1 and YTHDF2, disrupting their interaction with RNA in cells.^230^ Subsequent development of ebselen‐based structural analogues demonstrated the feasibility of targeting the YTH domain in YTHDF proteins, opening new avenues for developing factors that interfere with m6A recognition. Recent studies on the small molecule inhibitor DC‐Y13‐27, targeting YTHDF2, indicate it can enhance tumour response to radiotherapy and immunotherapy, providing a novel therapeutic strategy for cancer treatment.^188^ Li et al.^173^ designed an exosome‐mediated CRISPR/Cas9 plasmid DNA to target oncogenic YTHDF1 in vivo, contributing to YTHDF1 depletion and anti‐tumour activity. This approach offers a promising method to inhibit tumour proliferation by reactivating immune surveillance against tumours, though additional refinement in the delivery of the CRISPR system is still needed.^173^ M6A RNA demethylase inhibitors have shown potential in enhancing immune therapy,^231^, ^232^ and m6A methyltransferases METTL3^128^ and METTL14^233^ also exhibit anti‐tumour functions by reprogramming macrophage behaviour. Excitingly, our research suggests that combining immunotherapy with newly developed YTH domain inhibitors holds great potential for cancer treatment. YTH domain inhibitors, by regulating m6A modifications, can significantly enhance cancer response and sensitivity to immunotherapy. Specifically, enhancing m6A modifications can improve tumour cell recognition by the immune system, thereby strengthening the effects of immunotherapy. Enhancing m6A modifications improves tumour cell recognition by the immune system, strengthening immunotherapy effects, while inhibiting m6A modifications can weaken TIE mechanisms, increasing sensitivity to treatments like ICIs. Additionally, combining YTH domain inhibitors with existing chemotherapy and radiotherapy methods can further improve therapeutic outcomes. In certain types of cancer, the combination of YTH domain inhibitors and ICIs significantly improves patient prognosis. This discovery opens new possibilities for combination therapies, helping overcome the limitations of monotherapies. More research is essential to formulate more effective and selective inhibitors and activators of the YTH domain family through chemical synthesis, structure‐based virtual screening and screening of small molecule compound libraries. This research will also help determine the optimal types and doses of inhibitors and activators and optimise their combination with immunotherapy. These studies will elucidate the mechanisms of the YTH family in cancer treatment and provide new ideas and directions for developing more effective cancer therapies.

Targeting the YTH domain‐containing protein family in immunotherapy presents numerous challenges. Non‐specific targeting may impact normal cell functions, leading to toxicity and immune‐related adverse effects. Drug design is complex, requiring precise delivery to cancer cells while maintaining drug stability and bioavailability. Additionally, selecting appropriate targets and biomarkers, developing tools to detect YTH domain protein activity and monitoring therapeutic efficacy are crucial. Resistance is another significant issue. While YTH‐targeting drugs may overcome resistance to other medications, cancer cells might develop resistance to YTH inhibitors through mechanisms such as gene mutations. This necessitates further research and strategy development. Clinical trials are essential to verify safety and efficacy, and the challenges posed by individual differences require refined personalised treatment plans. Optimising combination therapy strategies and determining the best drug combinations and doses are also important. Despite these challenges, the potential of YTH domain proteins as therapeutic targets is immense. In‐depth research into their mechanisms in cancer, the development of precise and efficient drug designs and comprehensive clinical validation and optimisation can ultimately provide new treatment options for cancer patients.

## 8 CONCLUSIONS AND PROSPECTS

M6A modification has garnered significant attention in the life sciences due to its diverse functions. Biologists have focused on the dynamic interactions among m6A methyltransferases, m6A RNA demethylase inhibitors and m6A ‘readers’, such as the YTH protein family. It is suggested that m6A ‘readers’, including the YTH protein family, regulate immune responses and the TIME, linking the YTH protein family to immunotherapy. This review outlines the roles of the YTH protein family in immune regulation and as targets for anti‐tumour immunotherapy, summarising their mechanisms and recent developments. Although the exact molecular mechanisms of their regulation remain unclear, the YTH protein family is significantly associated with immune regulation. Complex immune mechanisms in tumour cells contribute to low response rates in immunotherapy. Therefore, the YTH protein family may be considered potential targets for immune‐related diseases and immunotherapy for tumours. Considering the numerous recent discoveries, it is necessary to update the academic progress related to the YTH protein family.

The dynamic and reversible nature of m6A modification makes it highly attractive in the field of anticancer tumour therapy. At present, it is crucial to identify the actual regulatory factors and understand their specific physiological roles in tumours, given the newly discovered physiological roles of m6A ‘readers’. This will aid in understanding the mechanism of action of the YTH protein family in immune therapy, enabling more accurate targeting of YTH proteins and providing fresh insights into strategies involving m6A modification and the YTH protein family in immune therapy. Furthermore, m6A modification exhibits cellular heterogeneity, with the same m6A methyltransferases, m6A RNA demethylase inhibitors and m6A ‘readers’ showing distinct biological roles in various cells. This heterogeneity could lead to opposing effects in tumour or immune‐related cells. Inhibitors of DNA and histone methylation have proven to enhance the success of immune therapy.^234^ Consequently, the ways in which m6A modification interfaces with DNA and histone epigenetics to control gene expression, as well as the potential associations between the YTH protein family and other types of methylation, remain unclear. Key questions include: How and when do YTH domain‐containing proteins participate in immune regulation? How do they interact with each other? Do YTH domain‐containing proteins act synergistically or antagonistically in different immune cells? Exploring whether combining YTH domain‐containing proteins with other epigenetic drugs can synergistically enhance immunotherapy is crucial. Additionally, specific YTHDF molecular inhibitors have yet to be developed. How can YTHDF be utilised in medical therapy? Are there other strategies to regulate YTHDF expression? These questions remain unresolved. Broader research on YTH domain family proteins in various immune cells and immune responses will pave the way for new therapeutic strategies involving YTH domains, including tumour immunotherapy, antiviral, anti‐inflammatory and autoimmune disease treatments. As this field evolves, it is crucial to accelerate clinical trials to translate theoretical findings into practical applications, thereby achieving significant progress in tumour immunotherapy, antiviral therapies, anti‐inflammatory treatments and autoimmune disease interventions.

In conclusion, the YTH protein family plays a crucial role in immune regulation and anti‐tumour immunotherapy through m6A modifications. These proteins regulate immune responses within the TIME and are involved in various immune checkpoint therapies, tumour resistance and immune‐related diseases. Targeting the YTH protein family can enhance the efficacy of immunotherapy and improve patient prognosis. However, developing efficient and selective YTH protein inhibitors and activators remains challenging and requires further research. Future studies should focus on elucidating the specific mechanisms of the YTH protein family, understanding their roles in immune regulation, developing combination therapies and validating their safety and efficacy through clinical trials to achieve breakthrough advancements in cancer therapy.

## AUTHOR CONTRIBUTIONS

L. F. H. conceived and designed the manuscript, wrote the main manuscript text and prepared the figures and tables. Z. C., L. J., W. L., Y. X. R. and Y. L. modified, reviewed the manuscript and figures. X. X. M. and H. W. conceived and designed the manuscript, acquisition of data, drafting of the article and final approval of the version. All authors read and approved the final manuscript.

## CONFLICT OF INTEREST STATEMENT

The authors declare that they have no conflict of interest.

## ETHICS STATEMENT AND CONSENT TO PARTICIPATE

Not applicable.

## CONSENT FOR PUBLICATION

Not applicable.

## Supporting information

Supporting Information

Supporting Information

Supporting Information

Supporting Information

## Data Availability

Not applicable.
