# Supplementary figures and images for: The YTH domain‐containing protein family: Emerging players in immunomodulation and tumour immunotherapy targets
Source: Clin Transl Med. 2024 Aug 12;14(8):e1784. doi: 10.1002/ctm2.1784 (PMC11319238; doi:10.1002/ctm2.1784)

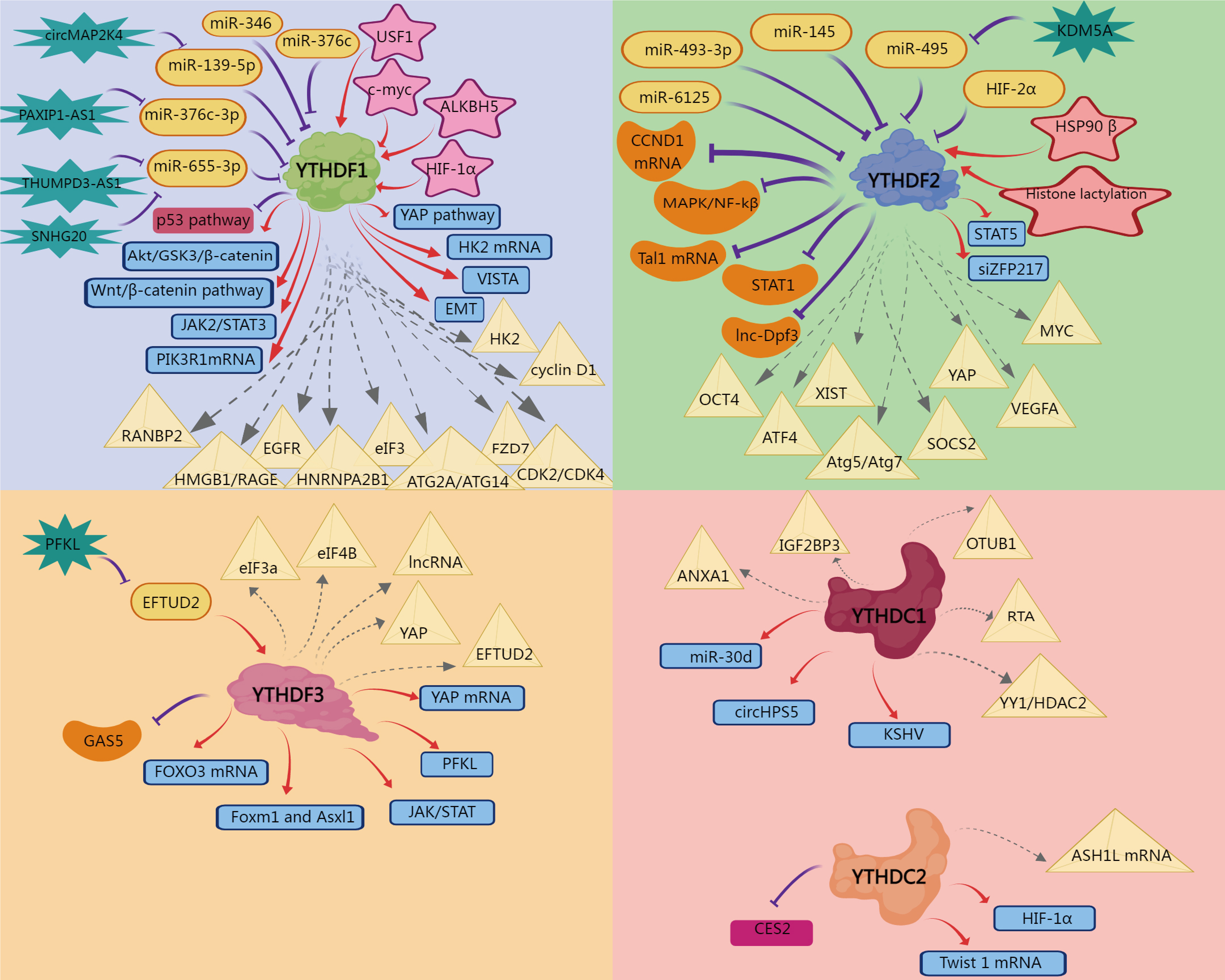

Supplement: Supplementary file 1 — Supporting Information [file CTM2-14-e1784-s003.jpeg]

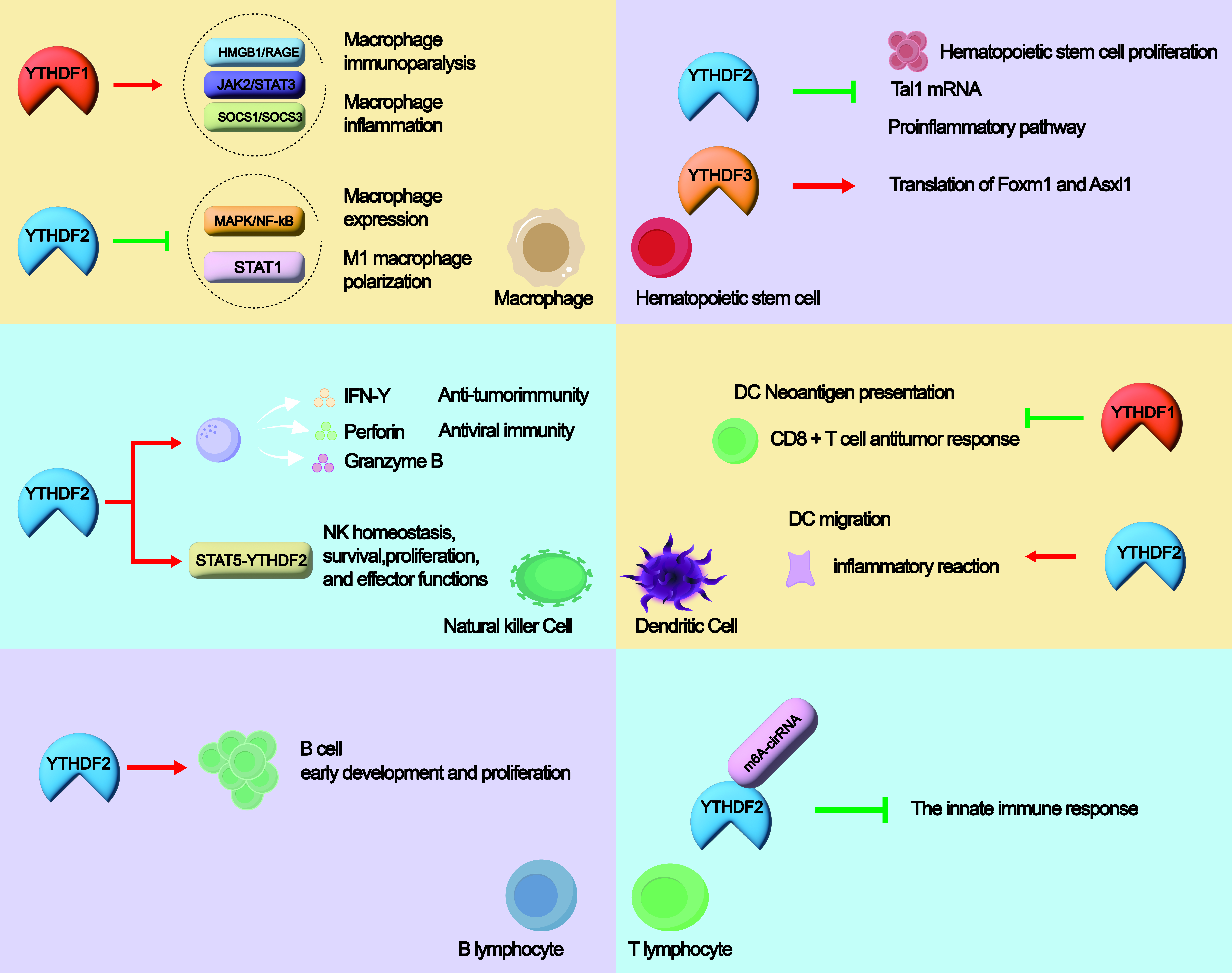

Supplement: Supplementary file 2 — Supporting Information [file CTM2-14-e1784-s002.jpeg]
