# Supplementary material for: The YTH domain‐containing protein family: Emerging players in immunomodulation and tumour immunotherapy targets
Source: Clin Transl Med. 2024 Aug 12;14(8):e1784. doi: 10.1002/ctm2.1784 (PMC11319238; doi:10.1002/ctm2.1784)
Supplement: Supplementary file 3 — Supporting Information [file CTM2-14-e1784-s004.docx]

**Supporting information 2| Table 1 Results for transcription factor binding sites in the predicted DNA sequences of the YTH domain-containing protein.**

| **Protein** | **Name [Matrix] Width** |
| --- | --- |
| YTHDF1 | C/EBPbeta [T00581] 4; YY1 [T00915] 4; RXR-alpha [T01345] 7; ER-alpha [T00261] 5; GR-alpha [T00337] 5; AP-2alphaA [T00035] 6; Pax-5 [T00070] 7; p53 [T00671] 7; TFII-I [T00824] 6; c-Myc [T00140] 6; FOXP3 [T04280] 6; GCF [T00320] 9; GR-beta [T01920] 5; PR B [T00696] 7; PR A [T01661] 7; NF-1 [T00539] 8; |
| YTHDF2 | C/EBPbeta [T00581] 4; IRF-2 [T01491] 6; GR-beta [T01920] 5; AP-2alphaA [T00035] 6; ER-alpha [T00261] 5; GR-alpha [T00337] 5; YY1 [T00915] 4; XBP-1 [T00902] 6; Pax-5 [T00070] 7; p53 [T00671] 7; TFIID [T00820] 7; TFII-I [T00824] 6; HNF-3alpha [T02512] 8; FOXP3 [T04280] 6; STAT4 [T01577] 6; STAT1beta [T01573] 10; c-Ets-1 [T00112] 7; WT1 [T00899] 9; GATA-1 [T00306] 6; TCF-4E [T02878] 7; GR [T05076] 7; RXR-alpha [T01345] 7; |
| YTHDF3 | YY1 [T00915] 4; GR-beta [T01920] 5; RXR-alpha [T01345] 7; GATA-1 [T00306] 6; C/EBPbeta [T00581] 4; GR [T05076] 7; STAT4 [T01577] 6; TFIID [T00820] 7; TFII-I [T00824] 6; FOXP3 [T04280] 6; AP-2alphaA [T00035] 6; AP-1 [T00029] 9; c-Jun [T00133] 7; c-Ets-1 [T00112] 7; Elk-1 [T00250] 9; Pax-5 [T00070] 7; p53 [T00671] 7; XBP-1 [T00902] 6; GR-alpha [T00337] 5; ER-alpha [T00261] 5; IRF-2 [T01491] 6; PR B [T00696] 7; PR A [T01661] 7; LEF-1 [T02905] 8; HNF-1A [T00368] 8; |
| YTHDC1 | C/EBPbeta [T00581] 4; YY1 [T00915] 4; GR-beta [T01920] 5; GR-alpha [T00337] 5; NF-Y [T00150] 8; ER-alpha [T00261] 5; TFIID [T00820] 7; HNF-3alpha [T02512] 8; GR [T05076] 7; TFII-I [T00824] 6; AP-2alphaA [T00035] 6; FOXP3 [T04280] 6; CREB [T00163] 9; IRF-2 [T01491] 6; GATA-1 [T00306] 6; RXR-alpha [T01345] 7; |
| YTHDC2 | STAT4 [T01577] 6; ER-alpha [T00261] 5; GR-beta [T01920] 5; C/EBPbeta [T00581] 4; GR-alpha [T00337] 5; TFII-I [T00824] 6; YY1 [T00915] 4; NF-1 [T00539] 8; Pax-5 [T00070] 7; GR [T05076] 7; FOXP3 [T04280] 6; p53 [T00671] 7; PR B [T00696] 7; PR A [T01661] 7; GATA-1 [T00306] 6; IRF-2 [T01491] 6; XBP-1 [T00902] 6; ATF-2 [T00167] 10; GCF [T00320] 9; HOXD9 [T01424] 10; HOXD10 [T01425] 10; HNF-3alpha [T02512] 8; TFIID [T00820] 7; |

Factors predicted within a dissimilarity margin less or equal than 0 % . Format Example: C/EBPbeta (Name), T00581 (Matrix)**,** 4 (Width).
