# Supplementary material for: The YTH domain‐containing protein family: Emerging players in immunomodulation and tumour immunotherapy targets
Source: Clin Transl Med. 2024 Aug 12;14(8):e1784. doi: 10.1002/ctm2.1784 (PMC11319238; doi:10.1002/ctm2.1784)
Supplement: Supplementary file 4 — Supporting Information [file CTM2-14-e1784-s001.docx]

**Supporting information 1| Figure 1 Expression regulation of relevant molecular targets of the YTH domain protein family.** The roles of five YTH family proteins, YTHDF1, YTHDF2, YTHDF3, YTHDC1, and YTHDC2, in cell signaling and gene expression regulation are shown. The purple section in the upper left corner illustrates the interactions of YTHDF1 with various miRNAs, proteins, and signaling pathways; the green section in the upper right corner displays the regulatory network of YTHDF2, including its interactions with miRNAs, proteins, and mRNAs; the yellow section in the lower left corner describes the relationship of YTHDF3 with transcription factors and signaling pathways; and the pink section in the lower right corner shows the interactions of YTHDC1 and YTHDC2 with miRNAs, viral proteins, and mRNAs. Each section highlights the crucial roles of YTH family proteins in cellular biological processes.

**Supporting information 1| Figure 2 Role of the YTH domain-containing protein family in immune cells.** The immune cells involved in the immune response include hematopoietic stem cells, macrophages, natural killer cells, dendritic cells, T lymphocytes, and B lymphocytes.
